# Absolute Bioavailability of Oxaliplatin After Intraperitoneal Administration by Electrostatic Pressurized Intraperitoneal Aerosol Chemotherapy (ePIPAC): Systemic Pharmacokinetics of the CRC-PIPAC-II Trial

**DOI:** 10.1245/s10434-025-18874-6

**Published:** 2026-01-25

**Authors:** Teun B. M. van den Heuvel, Paulien Rauwerdink, Emma Hulshof, Vincent C. J. van de Vlasakker, Dirk Jan A. R. Moes, Giulia Pluimakers, Koen P. B. Rovers, Geert-Jan Creemers, Pim J. W. A. Burger, Simon W. Nienhuijs, René J. Wiezer, Robin J. Lurvink, Djamila Boerma, Ignace H. J. T. De Hingh, Maarten J. Deenen

**Affiliations:** 1 Department of Oncology and Developmental Biology (GROW), Faculty of Health, Medicine and Life Sciences, Maastricht University, Maastricht, The Netherlands; 2 Department of Surgery, Catharina Hospital, Eindhoven, The Netherlands; 3 Department of Research & Development, Netherlands Comprehensive Cancer Organisation (IKNL), Utrecht, The Netherlands; 4 Department of Surgery, St. Antonius Hospital, Nieuwegein, The Netherlands; 5 Department of Clinical Pharmacy, Catharina Hospital, Eindhoven, The Netherlands; 6 Department of Clinical Pharmacy and Toxicology, Leiden University Medical Centre, Leiden, The Netherlands; 7 Department of Medical Oncology, Catharina Hospital, Eindhoven, The Netherlands

**Keywords:** Colorectal cancer, Peritoneal metastases, Palliative systemic chemotherapy, PIPAC, Pharmacokinetics, Bioavailability, Toxicity, Oxaliplatin

## Abstract

**Background:**

Pressurized intraperitoneal aerosol chemotherapy (PIPAC) offers a localized palliative treatment option for patients with colorectal peritoneal metastases (CPM), often combined with systemic therapy to maximize anti-tumor efficacy. This study on the pharmacokinetics of oxaliplatin-based PIPAC with electrostatic precipitation (ePIPAC-OX) aimed to determine the absolute bioavailability of oxaliplatin in plasma after ePIPAC-OX with reference to systemic therapy and to gain insights for optimizing therapy.

**Methods:**

This analysis included patients of the recently published CRC-PIPAC-II study, who received three cycles of oxaliplatin-based systemic therapy and ePIPAC-OX for unresectable CPM. Whole-blood and plasma ultrafiltrate samples were collected at five to six time points after both intravenous oxaliplatin and ePIPAC-OX. Pharmacokinetics were analyzed using population modeling. Absolute bioavailability was calculated as the fraction of the Area under the Curve (AUC0-∞) of the systemic oxaliplatin exposure after intraperitoneal administration over the AUC after intravenous administration, corrected for the dose.

**Results:**

The study included 18 patients, mostly treated with capecitabine and oxaliplatin (CAPOX) and bevacizumab (15 patients). The median dose-uncorrected AUC of systemic oxaliplatin and ePIPAC-OX in plasma was 165.2 μg*h/mL and 57.4 μg*h/mL, respectively. The median bioavailability of the total concentration and free fraction of oxaliplatin after ePIPAC-OX were both 48 % (interquartile range [IQR] 42–57 %). Dose reduction due to toxicity was required for eight patients (44 %). All the included patients experienced short-term symptoms of acute sensory neuropathy, with eight cases occurring after ePIPAC-OX.

**Conclusion:**

This is the first study to examine the absolute bioavailability of oxaliplatin administered by ePIPAC-OX in humans, using intra-patient data as a control measurement. The systemic bioavailability of oxaliplatin was substantial after ePIPAC-procedures. Therefore, ePIPAC-OX cannot be considered as solely a local treatment. Future research should take this into account for patients treated with both systemic chemotherapy and ePIPAC-OX as bidirectional therapy.

**Electronic supplementary material:**

The online version of this article (10.1245/s10434-025-18874-6) contains supplementary material, which is available to authorized users.

For approximately 10 % of colorectal cancer (CRC) patients, peritoneal metastases will be diagnosed during the course of their disease.^1^ About 10 % to 15 % of these patients will be eligible for curative-intent treatment through cytoreductive surgery and hyperthermic intraperitoneal chemotherapy (CRS-HIPEC). However, most patients with colorectal peritoneal metastases (CPM) (60–65 %) will undergo palliative treatment.^2^ These patients have a dismal prognosis, with a median overall survival (OS) of 12 months.^2^ This may be caused by the relative inefficacy of systemic therapy for patients with peritoneal metastases.^3–5^ A potential solution for this problem is by administration of the chemotherapy directly into the abdomen.^6,7^

Pressurized intraperitoneal aerosol chemotherapy (PIPAC) was developed to administer chemotherapy locally and minimally invasively as a pressurized aerosol.^8,9^ Generally, CPM patients are treated with oxaliplatin-based PIPAC (PIPAC-OX) (92 mg/m^2^) every 6 to 8 weeks. Additionally, electrostatic precipitation can be added to PIPAC, as studies have shown that this further enhances tissue penetration.^10,11^ This is referred to as ePIPAC. Furthermore, repetitive PIPAC-OX (either with or without electrostatic precipitation) can be combined with systemic therapy (i.e., bidirectional therapy). In bidirectional therapy, two different routes and mechanisms of administration are used. In this case, the underlying hypothesis for the combination with systemic chemotherapy is that this approach maximizes anti-tumor activity both systemically and locally at the tumor site.

Previously, a single-arm, phase 2 study (CRC-PIPAC) investigated repetitive electrostatic PIPAC-OX (ePIPAC-OX) as palliative monotherapy for 20 CPM patients after they had received different lines of palliative systemic treatment.^9^ This was the first study to describe the systemic pharmacokinetics after ePIPAC-OX. Surprisingly, despite the localized approach of PIPAC, pharmacokinetic outcomes from the CRC-PIPAC study showed that the systemic absorption of oxaliplatin after ePIPAC-OX was substantial.^12^ The combination of systemic chemotherapy and systemic exposure after ePIPAC-OX formed a risk for systemic toxicity. However, this study design did not allow for determining the absolute bioavailability of oxaliplatin after ePIPAC-OX.^12^

The recent CRC-PIPAC-II study evaluated the safety and efficacy of palliative first-line bidirectional therapy for 20 chemotherapy-naive patients with CPM.^13^ Further optimization of the therapeutic regimen remains crucial to maximize efficacy and reduce toxicity. Better understanding of the bioavailability of oxaliplatin is herein particularly important. Therefore, the current study aimed to determine the absolute bioavailability of oxaliplatin in plasma and plasma ultrafiltrate after administration via ePIPAC-OX.

## Methods

A prospective, single-arm, open-label, phase 2 study was performed in two Dutch tertiary referral centers. The United Medical Ethics Committee (MEC-U, Nieuwegein, the Netherlands) and the institutional review boards of both participating institutions approved the study (R19.087). The study was conducted in accordance with the principles of the Declaration of Helsinki. The full protocol plus clinical results have been published recently. Therefore, this report provides only a brief summary.^8,13^

### Patient Population

Patients were eligible for participation if they were at least 18 years old with a WHO performance score of 0-1, if they had a diagnosis of colorectal or appendiceal adenocarcinoma with unresectable peritoneal metastases, if they did not present with symptoms of obstruction, if they had adequate organ functions, if they did not have any contra-indications for chemotherapy or laparoscopy, if they had not received palliative chemotherapy for CRC, if they had not been treated with systemic chemotherapy within 6 months before inclusion, and if they had not undergone PIPAC previously. Informed consent was obtained from all patients.

### Bidirectional Therapy

**Fig. 1:**
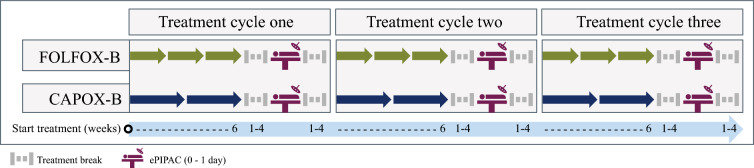
Study treatment design CRC-PIPAC-II trial according to oxaliplatin-based systemic regimens. This figure was adapted from Rauwerdink et al.^13^ The timeline indicates treatment in weeks, each treatment cycle consists of palliative systemic therapy alternated with electrostatic precipitation pressurized intraperitoneal aerosol chemotherapy (ePIPAC). Patients received either: FOLFOX (intravenous oxaliplatin [85mg/m^2^ BSA], leucovorin [400mg/m^2^ BSA], and 5-fluorouracil [400mg/m^2^ as bolus on day 1 followed by 46-hour continuous infusion of 2400mg/m^2^ BSA]) plus bevacizumab (5mg/kg on day 1) three courses every two weeks; or CAPOX (intravenous oxaliplatin [130mg/m^2^ BSA] on day 1, oral capecitabine [1000mg/m^2^ BSA] twice daily on days 1-14) plus bevacizumab (7.5mg/kg body weight on day 1) two courses every three weeks. ePIPAC encompasses intraperitoneal oxaliplatin (92mg/m^2^) with intravenous bolus of leucovorin (20mg/m^2^) and 5-fluorouracil (400mg/m^2^)

Patients received three cycles of first-line bidirectional therapy. Each cycle comprised 6 weeks of first-line systemic therapy followed by an ePIPAC-OX procedure. This procedure took place within 1 to 4 weeks after the intravenous administration of a cycle of systemic therapy, dependent on the regimen. Treatment with FOLFOX was after 1 to 2 weeks, and treatment with CAPOX was after 3 to 4 weeks. A schematic overview of the trial treatment is provided in Fig. 1. Trial treatment ended after the third ePIPAC-OX procedure regardless of disease response. Trial treatment could be terminated prematurely due to disease progression, toxicity, the patient’s request, or the physician’s discretion.

### First-Line Palliative Systemic Therapy

At the discretion of the treating medical oncologist, patients were treated with either CAPOX-bevacizumab, consisting of two 3-weekly cycles, or FOLFOX-bevacizumab, consisting of three 2-weekly cycles. The oxaliplatin was administered as a 2-h infusion. Doses are further specified in the legend of Fig. 1.

Notably, the CRC-PIPAC II trial allowed additional regimens that included irinotecan (i.e., FOLFIRI and FOLFOXIRI). However, to assess the bioavailability of oxaliplatin, this study focused only on those patients treated with oxaliplatin-based regimens.^13^

Type of regimen, dose reductions, switches between allowed regimens, and management of toxicity were left to the discretion of the treating medical oncologist. Dihydropyrimidine dehydrogenase (DPD; *DPYD*) status was assessed by *DPYD* genotyping before the first administration of systemic therapy, and dosages of capecitabine or 5-fluorouracil were modified accordingly.^14^

### ePIPAC-OX

The patients were treated with ePIPAC under general anesthesia with oxaliplatin (92 mg/m^2^) and a simultaneous intravenous bolus of leucovorin (20 mg/m^2^ in 10 min) and 5-fluorouracil (400 mg/m^2^ in 15 min). The intravenous bolus of leucovorin and 5-fluorouracil was infused during laparoscopic surgery, after which the intraperitoneal injection of oxaliplatin was started. Oxaliplatin was prepared in a total volume of 150 mL dextrose solution and injected through the nebulizer (CapnoPen; Capnomed GmbH, Zimmer ob Rottweil, Germany) in 5 min, after which the Ultravision generator (Ultravision, Alesi Surgical, Cardiff, UK) administered electrostatic precipitation to the aerosol. The electrostatic field and the 12-mm Hg capnoperitoneum were then maintained at 37 °C for another 25 min. The study protocol of both the CRC-PIPAC I and II trials provides a complete description of the ePIPAC-OX procedure.^8,15^

### Sample Collection and Analysis

The patients who underwent a complete first cycle of systemic therapy and successful ePIPAC-OX procedure were included for analysis. For each eligible patient, whole-blood samples were collected in heparin tubes at multiple time points during and after the first cycle of systemic therapy and the first ePIPAC-OX procedure. Thereby, each patient served as its own control measure, allowing precise determination of the bioavailability of oxaliplatin. The following sample collection schedules were adhered to:

1. CAPOX-bevacizumab: at *t* = 0, *t* = 0.5, *t* = 1, t=2 h and *t* = 3 weeks after the start of intravenous administration of oxaliplatin
2. FOLFOX-bevacizumab: at *t* = 0, *t* = 0.5, *t* = 1, t=2, *t* = 48 h and *t* = 2 weeks after the start of intravenous administration of oxaliplatin
3. ePIPAC-OX: at *t* = 0, *t* = 0.5, *t* = 1, *t* = 2 h and if patients were still admitted to the hospital at *t* = 16 hours and *t* = 1 week after intraperitoneal oxaliplatin injection.

Samples were directly cooled on ice and centrifuged to obtain plasma. One plasma aliquot was stored at −80 °C until further analysis for the total platinum concentration. A second aliquot with 1 mL of plasma was centrifuged through an ultrafiltrate Millipore membrane (Merck Millipore Ltd., Tullagreen, Carrigtwohill, Co. Cork, Ireland) for 20 min at 2000 g at 4 °C to obtain plasma ultrafiltrate and stored at −80 °C until analysis to determine the free fraction of oxaliplatin. Oxaliplatin concentrations were measured using atomic absorption spectrometry performed with a Thermo Fisher Solaar ICE 3500 graphite-furnace spectrophotometer with Zeeman correction (Thermo Fisher Scientific; Bremen, Germany) as described previously.^12^

### Population Pharmacokinetic Analysis

Concentration versus time data from both plasma and ultrafiltrate oxaliplatin were available for development of a population pharmacokinetic model. Data from both the plasma and ultrafiltrate compartment were modeled simultaneously. All estimations and simulations were performed using the nonlinear mixed-effects modeling software package, NONMEM V7.4.4 (Development Solutions, Ellicott City, MD, USA). One- and two-compartment models were explored as a structural model. The absorption rate constant was fixed to the value of the previously developed NONMEM model.^12^ Furthermore, an integrated two-compartment pharmacokinetic model for total and unbound oxaliplatin pharmacokinetics was explored. Inter-individual variability was assumed to be log-normally distributed. Additive, proportional, and combined error models were tested for residual variability in drug concentrations. Inter-individual variation was tested for all pharmacokinetic parameters. The structural model selection was based on reduction of the objective function value (OFV) (approximation of a chi-square distribution for nested models, with a ΔOFV of 3.84 corresponding to a *P* value of 0.05), goodness-of-fit (GOF) plots, shrinkage, and precision of pharmacokinetic parameter estimates.

Individual predicted (IPRED) and observed concentrations as well as population predictions (PRED) and observed concentrations (DV) for oxaliplatin pharmacokinetics in plasma and ultrafiltrate were used to create GOF plots. These plots included IPRED versus DV, PRED versus DV, conditional weighted residual error (CWRES, which is the weighted difference between the model prediction based on estimation approximation and the real data^16^) versus PRED, and CWRES versus time after last dose (TAD). The final model was subsequently evaluated using GOF plots and 500 prediction-corrected visual predictive checks (pcVPCs). The Perl-speaks-NONMEM tool kit version 4.7.0 (Uppsala, Sweden) and Pirana version 2.9.7 (Denekamp, Netherlands) were used as the modeling environment. Results were plotted using R statistics (v3.4.4; Boston, MA, USA) and RStudio (v1.1.453; Boston, MA, USA). The first-order conditional estimation method with interaction was used throughout the analysis.

### Calculation of Absolute Bioavailability

Finally, the area under the curves (AUCs) of oxaliplatin after intravenous administration and intraperitoneal administration were estimated using the final population pharmacokinetic model. The absolute bioavailability (F_abs_) was calculated as the fraction of the AUC_0-∞_ of the systemic oxaliplatin exposure after intraperitoneal administration over the AUC after intravenous administration, corrected for the dose^17^:

$${\mathrm{F}}_{{{\mathrm{abs}}}} = { 1}00 \, \% \, \times \, \left( {{\mathrm{AUC}}_{{{\mathrm{i}}.{\mathrm{p}}. \, 0 - \infty }} \times {\text{ dose}}_{{{\mathrm{i}}.{\mathrm{v}}.}} } \right)/\left( {{\mathrm{AUC}}_{{{\mathrm{i}}.{\mathrm{v}}}} ._{0 - \infty } \times {\text{ dose}}_{{{\mathrm{i}}.{\mathrm{p}}.}} } \right).$$

Based on the acquired data and developed NONMEM model, a simulation was performed on the oxaliplatin concentration over time after intravenous and intraperitoneal administration.

## Results

### Patient Characteristics

Between February 2020 and August 2021, 20 patients were treated with palliative systemic therapy and repetitive ePIPAC-OX. A total of 18 patients received oxaliplatin-based systemic regimens and could be included in this pharmacokinetic side study. Two patients were not considered eligible: the one patient received FOLFIRI as systemic therapy, and the other patient was excluded because of logistics during the ePIPAC-procedure, making pharmacokinetic sampling ineligible. Baseline demographics and disease characteristics of the included 18 patients are presented in Tables 1, 2 and 3.

**Table 1:** Patient characteristics

Characteristic	Category	*n*	%
Gender	Male	8	44
Age (years)	Median (range)	54 (42–70)	
Location of primary tumor	Appendix	8	44
	Ascending colon	8	44
	Descending colon	2	11
Histology of primary tumor	(mucinous) adenocarcinoma	11	61
	(mucinous) adenocarcinoma with SRC	4	22
	Goblet cell carcinoma LAMN	1 2	6 11
Primary tumor resection	Yes	4	22
Diagnosis of PM	Synchronous	15	83
Previous systemic treatment CRC Palliative systemic therapy Initial PCI^c^	None	18	100
	Neoadjuvant therapy Adjuvant therapy	0 0	
	CAPOX ^a^–bevacizumab FOLFOX ^b^–bevacizumab Median (range)	15 3 34 (24–39)	83 17

*SRC*, signet ring cell; *LAMN*, low-grade appendiceal mucinous neoplasm; *PM*, peritoneal metastases; *CRC*, colorectal cancer; *PCI*, Peritoneal Cancer Index. ^a^capecitabin + oxaliplatin; ^b^5-Fluoruracil + oxaliplatin; ^c^as determined during first PIPAC procedure

**Table 2:** Pharmacologic traits and pharmacokinetic results of the total platinum exposure per patient

ID Unit	Dose systemic (mg)	AUC systemic (μg*h/mL)	AUC systemic free fraction (μg*h/mL)	Dose PIPAC (mg)	AUC PIPAC (μg*h/mL)	AUC PIPAC free fraction (μg*h/mL)	Clearance^*a*^ (mL/min.)	Bioavailability^*a*^ (%)
1	300	185.9	45.0	185	57.8	14.0	1.61	50.4
2	270	193.5	42.4	185	55.8	12.2	1.40	42.1
3	205	163.0	28.4	145	72.1	12.6	1.26	62.5
4	265	184.2	44.0	185	61.3	14.6	1.44	47.7
5	230	176.1	36.5	160	66.2	13.7	1.31	54.1
6	245	147.1	27.2	170	57.1	10.6	1.67	55.9
7	265	175.8	28.5	185	47.9	7.8	1.51	39.0
8	150	136.3	27.6	165	40.0	8.1	1.10	26.7
9	240	207.5	39.9	165	63.2	12.1	1.16	44.3
10	170	122.9	18.1	165	46.3	6.8	1.38	38.9
11	190	106.8	17.6	180	40.6	6.7	1.78	40.2
12	275	166.7	27.9	180	68.9	11.5	1.65	63.1
13	230	176.3	28.6	180	93.2	15.1	1.30	67.5
14	230	162.6	26.8	160	55.3	9.1	1.41	48.8
15	220	152.5	25.0	160	65.3	10.7	1.44	58.9
16	325	215.6	46.1	180	57.0	12.2	1.51	47.8
17	170	107.8	14.8	180	52.0	7.1	1.58	45.5
18	250	163.6	30.6	170	63.1	11.8	1.53	56.7
Median (IQR)	235 (201–266)	165.2 (144.4–184.6)	28.5 (26.3–40.5)	175 (164–181)	57.4 (50.9–65.5)	11.7 (8.0–12.9)	1.44 (1.31–1.59)	48.3 % (41.6–57.2)
*Geometric mean (*coefficient of variance): *n* (%)		160.6 (19)	29.4 (31)		57.9 (21)	10.6 (25)	1.44	48.3

*AUC*, area under the curve; *PIPAC*, pressurized intraperitoneal aerosol chemotherapy; *IQR*, interquartile range. ^a^Clearance and bioavailability are independent of route of administration

The median age was 54 years (range, 42–70 years), and 56 % of the patients were female. The patients presented with extensive peritoneal disease, as the median peritoneal cancer index (PCI) score was 34 (range, 24–39). Adenocarcinoma was most prevalent (11 patients; 61 %), as well as right-sided tumors (8 appendix and 8 ascending colon tumors, both 44 %). The majority of the patients had synchronous disease (15 patients, 83 %) and no prior resection of the primary tumor (14 patients, 78 %). All the patients were chemotherapy-naïve at the time of inclusion, and no renal or hepatic disease was present. Most of the patients (15 patients; 83 %) received systemic treatment with CAPOX and bevacizumab. Altogether, 72 % of systemic cycles were administered without any dose reductions. Dose reductions of systemic oxaliplatin due to toxicity were required for nine patients (50 %). For the patients who needed dose reduction, the reductions were executed in median cycle number 4. Capecitabine dose reductions were required for nine patients, whereas no dose reductions of ePIPAC-OX were required in the total study population. All the included patients experienced symptoms of acute sensory neuropathy during treatment. In eight patients, acute sensory neuropathy also appeared after their ePIPAC-procedure.

### Oxaliplatin Pharmacokinetics

**Fig. 2:**
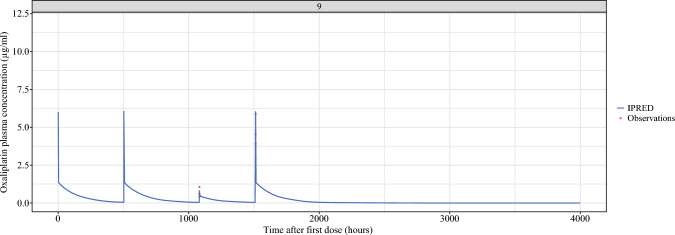
Free oxaliplatin plasma concentration model over time, after both systemic therapy and ePIPACOX; example of one patient. The higher peaks (first, second and fourth) are a result of systemic treatment, while the smaller peak (the third) indicates the oxaliplatin plasma concentration after ePIPAC-OX. IPRED: individual prediction

**Fig. 3:**
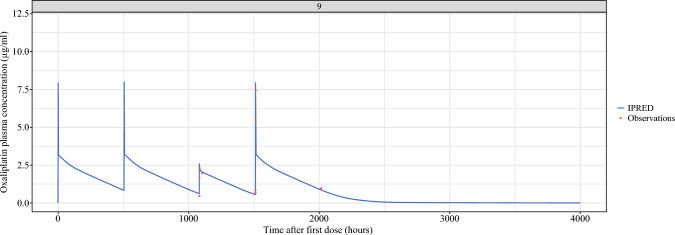
Total oxaliplatin plasma concentration model over time, after both systemic therapy and ePIPACOX; example of one patient. The higher peaks (first, second and fourth) are a result of systemic treatment, while the smaller peak (the third) indicates the oxaliplatin plasma concentration after ePIPAC-OX. IPRED: individual prediction

Figures 2 and 3 illustrate the pharmacokinetic profiles of oxaliplatin over time in plasma and plasma ultrafiltrate in a typical patient. Pharmacokinetic profiles of the other patients are depicted in the the Supplementary Data. The median dose-uncorrected AUC of systemic oxaliplatin in plasma was 165.2 μg*h/mL (interquartile range [IQR], 144.4–184.6 μg*h/mL). The median dose-uncorrected AUC of ePIPAC-OX in plasma was 57.4 μg*h/mL (IQR, 50.9–65.5 μg*h/mL). When corrected for the administered dose, the median bioavailability of the total oxaliplatin concentration after peritoneal administration was 48 % (IQR, 42–57 %).

Regarding systemic exposure of the free (ultrafiltrate) fraction of oxaliplatin, the median dose-uncorrected AUC of intravenous oxaliplatin was 28.5 μg*h/mL (IQR, 26.3–40.5 μg*h/mL). The median dose-uncorrected AUC of the free fraction after ePIPAC-OX was 11.7 μg*h/mL (IQR, 8.0–12.9 μg*h/mL). Because a fixed equilibrium exists between the protein-bound and unbound (free) fractions for a given drμg, the bioavailability for both the total and unbound fraction are similar and had for both the total and the free fraction a median bioavailability of 48 % (IQR, 42–57 %).

Despite the similar bioavailability, the total maximum concentration (C_max_) differed between the two routes of administration, as the median C_max_ was 7.6 μg/mL (IQR, 6.3–8.7 μg/mL) for systemic administration and 3.6 μg/mL (IQR, 2.6–3.1 μg/mL) for PIPAC. The median time after which this total C_max_ was reached (T_max_) was 122 min (IQR, 120–127 min) for systemic administration and 85 min (IQR, 72–134 min) after PIPAC. The median C_max_ values for the free (ultrafiltrate) fraction for systemic administration and PIPAC were 3.44 μg/mL (IQR, 2.62–3.90 μg/mL) and 1.45 μg/mL (IQR, 1.31–1.98 μg/mL), respectively, with corresponding T_max_ times of 120 min (IQR, 106–124 min) and 65 min (IQR, 33–71 min), respectively. Table 2 shows the AUC, dose, clearance, and bioavailability for all the individual patients, whereas the measurements of C_max_ and corresponding T_max_ for all four groups (total concentration and ultrafiltrate after systemic administration and PIPAC) are presented in Table 4.

**Fig. 4:**
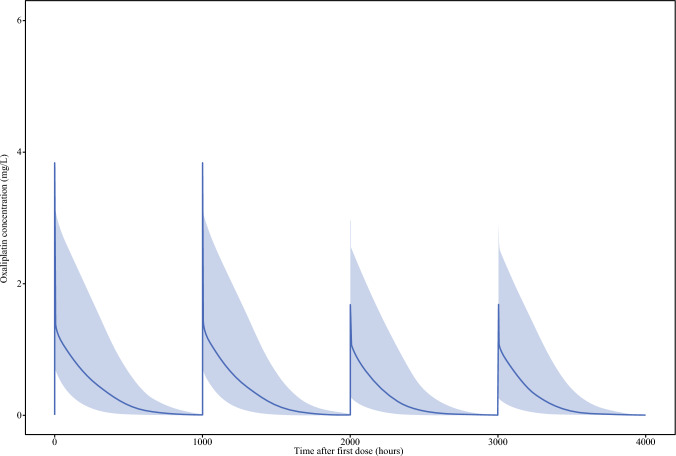
Simulation of oxaliplatin concentration over time after systemic cycle and ePIPAC-OX procedure

Figure 4 plots the concentration-time curves of two simulated doses of intravenous oxaliplatin (160 mg) and two simulated doses of ePIPAC-OX (160 mg).

### POPPK Modeling Oxaliplatin

**Fig. 5:**
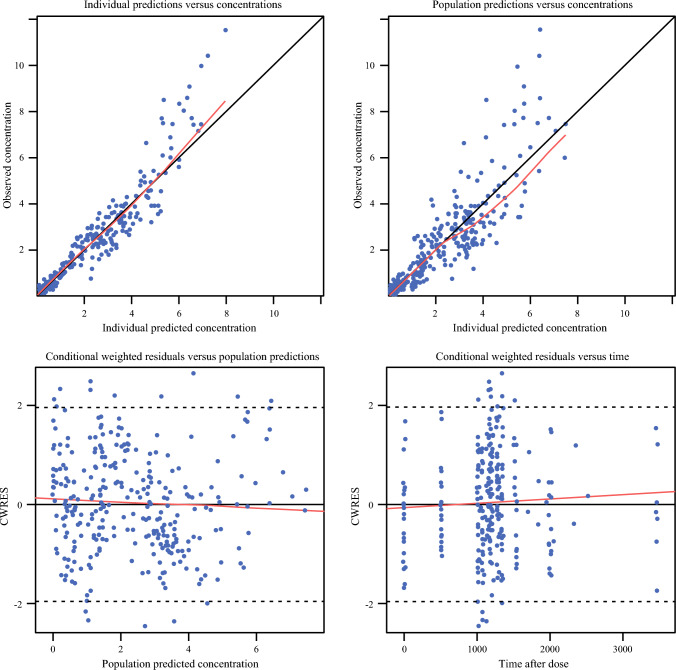
Goodness of fit plots for population pharmacokinetics (POPPK) oxaliplatin model

The pharmacokinetics of oxaliplatin were best described by an integrated two-compartment pharmacokinetic model for total and unbound oxaliplatin pharmacokinetics. The model code is provided in the Appendix. The final population pharmacokinetic parameter estimates are presented in Table 5. The goodness of fit is presented in Fig. 5. These indicate that the observed concentrations were adequately described.

## Discussion

This study aimed to determine the absolute bioavailability of oxaliplatin after administration by ePIPAC-OX for patients with unresectable peritoneal metastases of a colorectal origin. The results show that the absolute systemic bioavailability of intraperitoneal oxaliplatin administered by ePIPAC is 48 % after the first ePIPAC procedure. To the best of our knowledge, this is the first in-human study to assess the absolute bioavailability of a chemotherapeutic agent administered by ePIPAC, making it unique evidence on the bioavailability of oxaliplatin after ePIPAC-OX. It clearly shows that ePIPAC-OX may not entirely be considered a local treatment because it leads to significant systemic exposure.

### Bioavailability

In the current study, whole-blood samples were acquired both during and after the first systemic treatment of a cycle and ePIPAC-OX. As a result, the absolute bioavailability of oxaliplatin after ePIPAC-OX could be precisely determined within patients because the patients directly served as their own controls because they also received an intravenous oxaliplatin infusion.

The results on pharmacokinetics from patients in the CRC-PIPAC-I trial and in the current study were combined and analyzed to acquire a robust model that best describes the bioavailability in patients. Analysis of the measurements resulted in a median bioavailability of 48 % (IQR, 42–57 %) for the total amount of oxaliplatin. In other words, almost half of the oxaliplatin administered through ePIPAC-OX is absorbed systemically. Our findings are in line with previous findings in patients treated with HIPEC, in which a study assessing systemic exposure also found that the mean absorbed amount of oxaliplatin after HIPEC was 48 %.^18^

Both HIPEC and PIPAC have traditionally been regarded as local treatments, aiming to minimize systemic exposure. This reduced exposure is expected to result in less systemic toxicity, which is considered one key advantage of both the HIPEC and PIPAC treatments.^19^ However, oxaliplatin is known to have a relatively lower AUC ratio of intraperitoneal-to-plasma concentration (AUC, 16) than other intraperitoneal chemotherapy agents such as mitomycin C (AUC, 23.5), doxorubicin (AUC, 230), 5-FU (AUC, 250) and taxane drugs (AUC, ±1000). A large intraperitoneal-to-plasma ratio means that high concentrations within the abdomen are maintained, whereas a lower ratio indicates a relatively higher systemic absorption into the systemic circulation.^20^ Both cohorts supported the observation that a significant part of ePIPAC-OX ends up systemically as well.

### Clinical Interpretation

Acute sensory neuropathy is one of the most common side effects of systemic treatment with oxaliplatin, occurring in 70 % of patients.^21^ Notably, all the patients (100 %) in this study experienced (short-term) symptoms of this acute sensory neuropathy during study treatment, with eight patients experiencing these symptoms directly after the ePIPAC-procedure. This might suggest that alternating systemic oxaliplatin with ePIPAC-OX, a bidirectional approach, causes an accumulation of oxaliplatin, possibly with toxicity as a result. Other observed treatment-related complications included mainly reversible abdominal pain, nausea, and fatigue. However, it remains difficult to determine whether these complaints are caused by the systemic therapy, the ePIPAC, the disease, or a combination of these factors.^13^

Regardless of the exact degree of impact of the ePIPAC, the main message of the current study is that ePIPAC-OX impacts the systemic bioavailability of oxaliplatin. As a result, it may be of influence for patients in whom toxicity occurs because the systemic bioavailability could increase the risk of (cumulative) peripheral sensory neuropathy (PNP). Thus, clinicians should take this potential impact of ePIPAC into consideration when cumulative PNP or any other form of toxicity occurs.

Using electrostatic precipitation as part of the PIPAC procedure ensures a faster and larger tissue uptake after intraperitoneal delivery than a regular PIPAC procedure.^10,22,23^ Earlier, this was deemed an advantage of the ePIPAC-procedure. However, as the current study showed, the peritoneum is exposed to oxaliplatin for only a limited time as a result of the usage of electrostatic precipitation. Moreover, this trait influences the increased passage of a cytostatic agent across the plasma-peritoneum barrier, and thus the increased systemic exposure. Thus, electrostatic precipitation in the ePIPAC procedure may potentially cause both an increased systemic exposure and a decreased duration of peritoneal exposure, which are key aspects clinicians aim to prevent when using bidirectional therapy.

Other aspects that might affect systemic absorption from the peritoneum are the extensiveness and characteristics of the peritoneal disease. A previous study showed that a higher PCI score increases the permeability of the peritoneal barrier, most likely explained by the presence of inflammation.^24^ This would mean that patients with more extensive disease, which is the case for patients who receive PIPAC, may have an increased systemic exposure after intraperitoneal treatment. This observation is in line with the previous pharmacokinetic study in the CRC-PIPAC phase 1 trial, which found that the oxaliplatin concentrations in the peritoneum 30 min after the start of the ePIPAC procedure decreased during three sequential ePIPAC-OXs, whereas the systemic exposure of oxaliplatin in the second and third ePIPAC-OX increased by approximately 20 % compared with the first ePIPAC-OX.^12^

### Intraperitoneal Treatment

To date, only animal studies on the absolute bioavailability have been performed. A study on oxaliplatin-based PIPAC in mice concluded that the systemic passage of oxaliplatin in blood was lower after PIPAC compared with intravenous administration alone. Simultaneously, the peritoneal response was similar, indicating both a lower needed dose and a decreased risk of toxicity after PIPAC.^25^

Another anticancer drug used in peritoneal treatment is irinotecan, of which SN-38 is the active metabolite. One study examined the exposure to active SN-38 after intraperitoneal administration of irinotecan in pigs. Although this study did not evaluate the administration through PIPAC, it did provide information on intraperitoneal treatment. The study concluded that it is possible to acquire a peritoneal exposure to SN-38 that is 30 times higher than the systemic exposure. Nonetheless, toxicity, mainly of a gastrointestinal nature, occurred in these pigs.^26^ These previous studies emphasize that toxicity is a relevant issue when intraperitoneal treatment is used, just as the current study concluded.

### PIPAC Versus Catheter-Based Intraperitoneal Chemotherapy

The aspect of intra)peritoneal exposure is being further investigated. In the Netherlands, a clinical trial on catheter-based intraperitoneal chemotherapy currently is ongoing.^27^ In this trial, patients with unresectable peritoneal metastases of a colorectal origin are treated with a combination of systemic modified FOLFOX4 and intraperitoneal irinotecan.^27^ By combining different types of chemotherapy, potential accumulation of oxaliplatin, for example, is prevented. The results of this phase 2 trial are still awaited, but if systemic bioavailability and toxicity appear to be lower in these patients, this may be an important factor to take into consideration when deciding which bidirectional therapy should be favored.

### Study Limitations

The results of this study were limited by the size of the study population, as only 18 patients were included. As a result, a small number of measurements could be performed and analyzed. However, as can be seen in the results, the interquartile range was narrow. In addition, for pharmacokinetic purposes, a sample size of 18 generally is considered adequate.

Furthermore, in this study, only whole-blood samples were acquired and not peritoneal tissue concentrations. Information on tissue concentrations may be interesting as well because it would provide further knowledge on the distribution of the administered oxaliplatin after an ePIPAC-procedure.

The current data were acquired using patients who underwent ePIPAC-procedures. Therefore, no definitive conclusions can be drawn on regular PIPAC without electrostatic precipitation. Nonetheless, the data of the current study imply that a substantial part of the intraperitoneally administered chemotherapy becomes systemically available.

Finally, oxaliplatin is known to have a long half-life, which leads to an incomplete washout between intravenous oxaliplatin and intraperitoneal oxaliplatin.^28^ However, by using the previously described model, it was possible to correct for this and to estimate the AUC accurately.

## Conclusion

This is the first study to examine the absolute bioavailability of oxaliplatin administered by ePIPAC-OX in humans while using unique intra-patient data as control measurement. Systemic bioavailability of oxaliplatin was shown to be substantial after ePIPAC procedures, which contradicts one of the hypothesized advantages of PIPAC. Thus, ePIPAC-OX cannot be considered solely as a local treatment. This should be taken into account when toxicity occurs in patients treated with both systemic chemotherapy and ePIPAC-OX as bidirectional therapy.

## Electronic supplementary material

Below is the link to the electronic supplementary material.

Supplementary file 1 (PDF 374 kb)

Supplementary file 2 (PDF 335 kb)

## Figures and Tables

**Figure:**
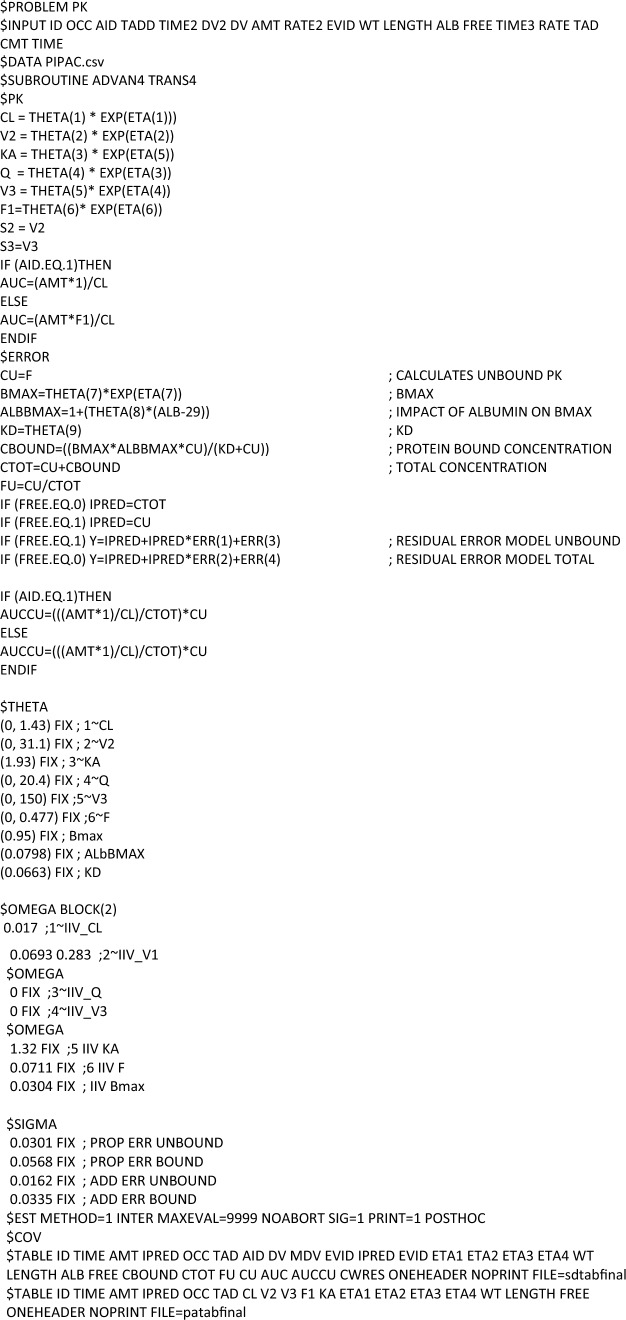
Final model code

## Appendix

See Tables 3, 4, 5

**Table 3:** Table on all patient characteristics (adapted from Rauwerdink et al.^14^)

ID	Sex	Age (yrs)	Primary tumor location	Histology	Onset of PM	Time diagnosis PM to (weeks):	
						Enrollment	First ST
1	Male	41	Ascending colon	AC	Metachronous	5	8
2	Male	54	Appendix	LAMN ^a^	Synchronous	5	6
3	Female	70	Transverse colon	AC-SRC	Synchronous	7	10
4	Male	61	Ascending colon	MAC	Synchronous	3	4
5	Female	62	Appendix	AC	Synchronous	3	5
6	Male	54	Ascending colon	MAC	Synchronous	3	6
7	Male	57	Transverse colon	MAC	Metachronous	3	5
8	Female	68	Caecum	AC	Synchronous	5	7
9	Female	45	Appendix	GCC	Synchronous	4	5
10	Female	69	Appendix	MAC	Synchronous	6	8
11	Female	59	Caecum	MAC	Synchronous	1	2
12	Male	54	Sigmoid	MAC	Synchronous	3	5
13	Female	64	Appendix	AC-SRC	Synchronous	4	6
14	Male	54	Appendix	Unknown ^b^	Synchronous	5	7
15	Female	43	Appendix	MAC-SRC	Synchronous	8	9
16	Male	70	Ascending colon	MAC	Metachronous	6	8
17	Female	47	Sigmoid	AC	Synchronous	3	4
18	Female	45	Appendix	AC	Synchronous	8	9

This figure was adapted from Rauwerdink et al.^13^ *PM p*eritoneal metastases; *ST* systemic therapy; *AC* adenocarcinoma; *LAMN* low-grade appendiceal mucinous neoplasm; *SRC* signet ring cell; *MAC* mucinous adenocarcinoma; *GCC* Goblet Cell (adeno)carcinoma. ^a^Histopathological assessment of resected specimens unexpectedly revealed low-grade pseudomyxoma peritonei originating of a low-grade appendiceal mucinous neoplasm. ^b^Reviewed histopathology and unexpected diagnosis of high-grade pseudomyxoma peritonei originating from primary tumor of unknown histologic grade (in situ)

**Table 4:** Overview of outcomes of C_max_ and T_max_ (i.e. corresponding time to C_max_) for systemic administration and PIPAC

ID Unit	C_max_ total after systemic (ug/mL)	T_max_ total after systemic (minutes)	C_max_ UF after systemic (ug/mL)	T_max_ UF after systemic (minutes)	C_max_ total after PIPAC (ug/mL)	T_max_ total after PIPAC (minutes)	C_max_ UF after PIPAC (ug/mL)	T_max_ UF after PIPAC (minutes)
1	5.99	120	3.41	120	2.27	80	1.84	30
2	7.16	123	3.53	123	2.51	80	1.37	80
*3*	9.96	120	3.91	120	2.45	N/A	N/A	65
4	7.70	120	3.90	120	2.81	85	1.74	30
5	10.41	120	3.67	120	3.67	70	2.13	70
6	5.41	120	2.28	120	3.00	120	1.42	30
7	8.59	120	3.12	124	3.14	145	1.45	40
8	8.51	120	3.92	120	3.58	60	1.37	30
9	11.55	120	5.88	120	2.31	130	1.05	65
10	7.45	120	3.28	120	3.62	72	1.25	72
11	2.24	127	1.35	127	2.60	163	0.72	53
12	7.48	135	2.64	135	3.12	212	1.48	107
13	9.07	124	3.88	64	5.19	66	2.58	66
14	8.32	123	2.84	65	2.58	86	N/A	N/A
15	6.43	127	2.57	65	3.40	85	2.32	45
16	7.46	125	3.94	125	3.41	67	1.72	67
17	4.80	134	1.06	38	3.92	81	1.41	81
18	7.69	130	3.48	130	2.93	125	2.14	35
Median (IQR)	7.6 (6.3–8.7)	122 (120–127)	3.4 (2.6–3.9)	120 (106–124)	3.6 (2.6–3.1)	85 (72–134)	1.5 (1.3–2.0)	65 (33–71)

**Table 5:** Population pharmacokinetic parameters of the final model

	Mean value	RSE (%)	Shrinkage (%)
CL (L/hour)	1.44	6.7	
V_2_ (L)	31	12.1	
Ka (/hour)	1.93	Fixed	
Q (L/hour)	20,4.	11.2	
V3 (L)	150	9.2	
F	0.477	9.9	
Bmax (µmol/L)	0.95	Fixed	
AlbBmax	0.0798	10.7	
Kd (µmol/L)	0.0633	Fixed	
Interindividual variability (IIV)			
CL (CV%)	13	19.9	3
V2 (CV%)	53.2	15	3
Cl~V2 (CV%)	101.1%		
Ka (CV%)	114.9	19	18
F (CV%)	26.7	29	16
Bmax (CV%)	17.5	20	20
Random residual variability			
Unbound Proportional (%)	17.3	23	13
Bound Proportional (%)	23.8	20	5
Unbound Additive (ug/mL)	0.1	29	13
Bound Additive (ug/mL)	0.2	30	5

*RSE* relative standard error, *CL* clearance, *V2* volume of distribution central compartment, Q inter compartmental clearance, *V3* volume of distribution of peripheral compartment, *F* Bioavailability, *Bmax* maximum binding capacity, *AlbMax* impact of albumin on Bmax, *Kd* is the equilibrium dissociation constant., *CV%* percentage coefficient of variation
