# Supplementary figures and images for: Absolute Bioavailability of Oxaliplatin After Intraperitoneal Administration by Electrostatic Pressurized Intraperitoneal Aerosol Chemotherapy (ePIPAC): Systemic Pharmacokinetics of the CRC-PIPAC-II Trial
Source: Ann Surg Oncol. 2026 Jan 25;33(4):3004–17. doi: 10.1245/s10434-025-18874-6 (PMC12982276; doi:10.1245/s10434-025-18874-6)

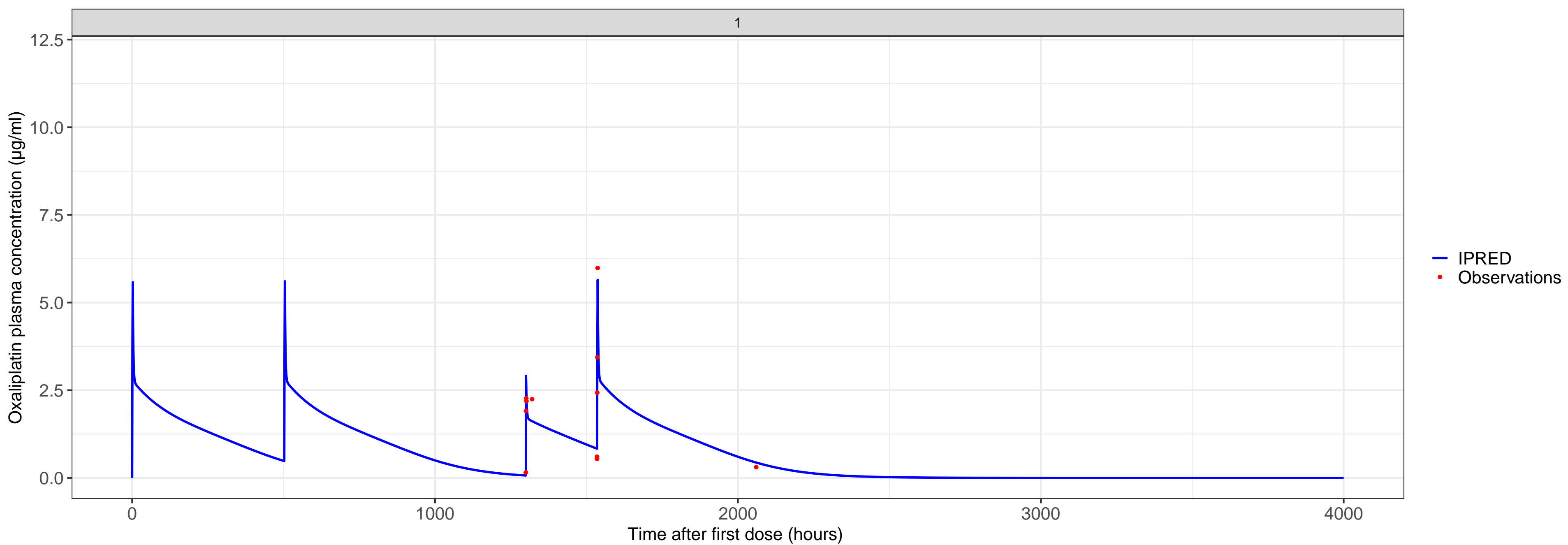

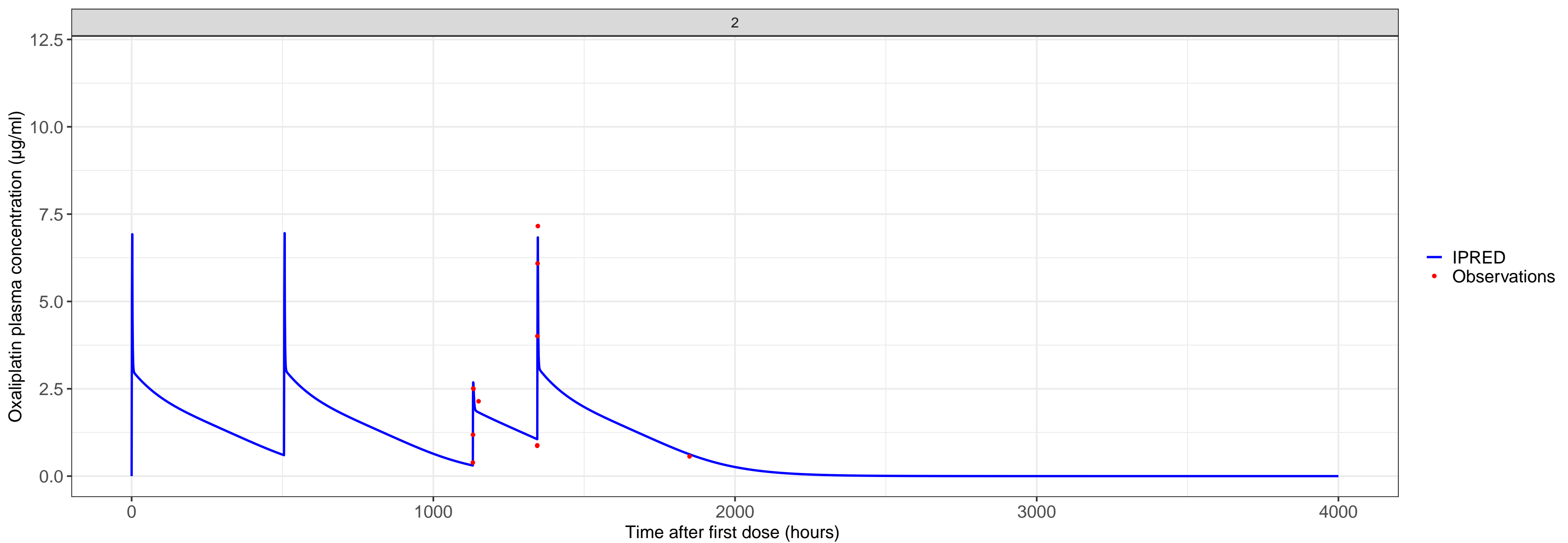

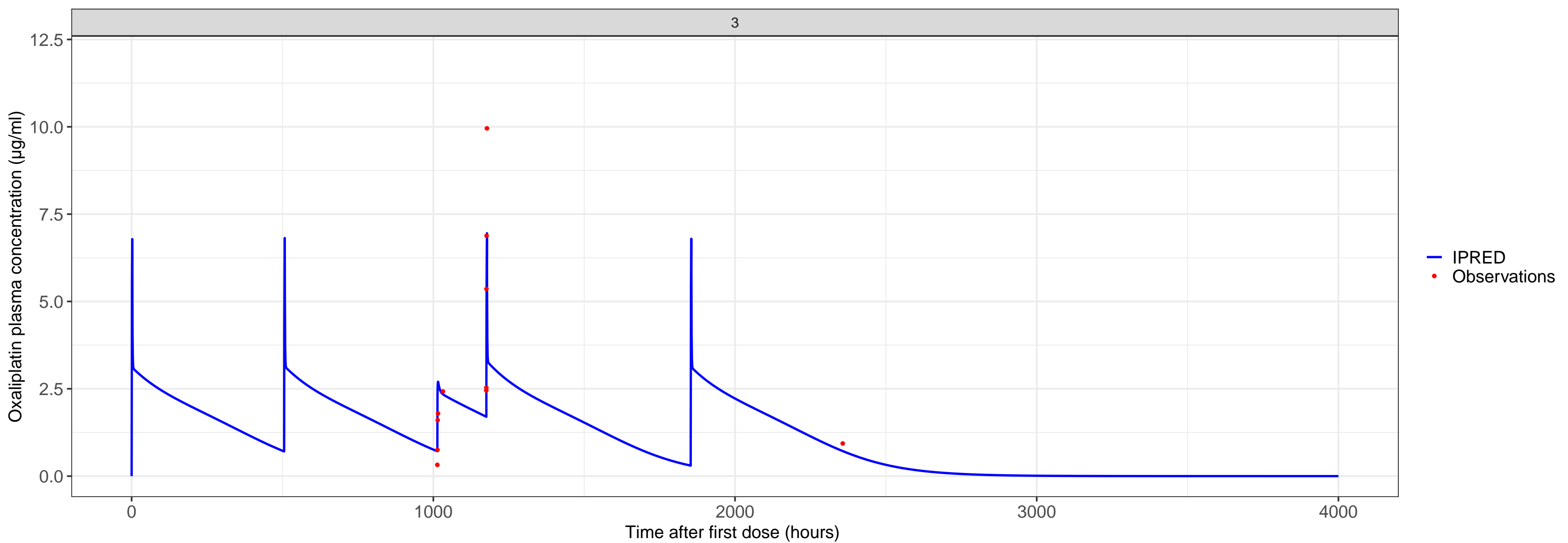

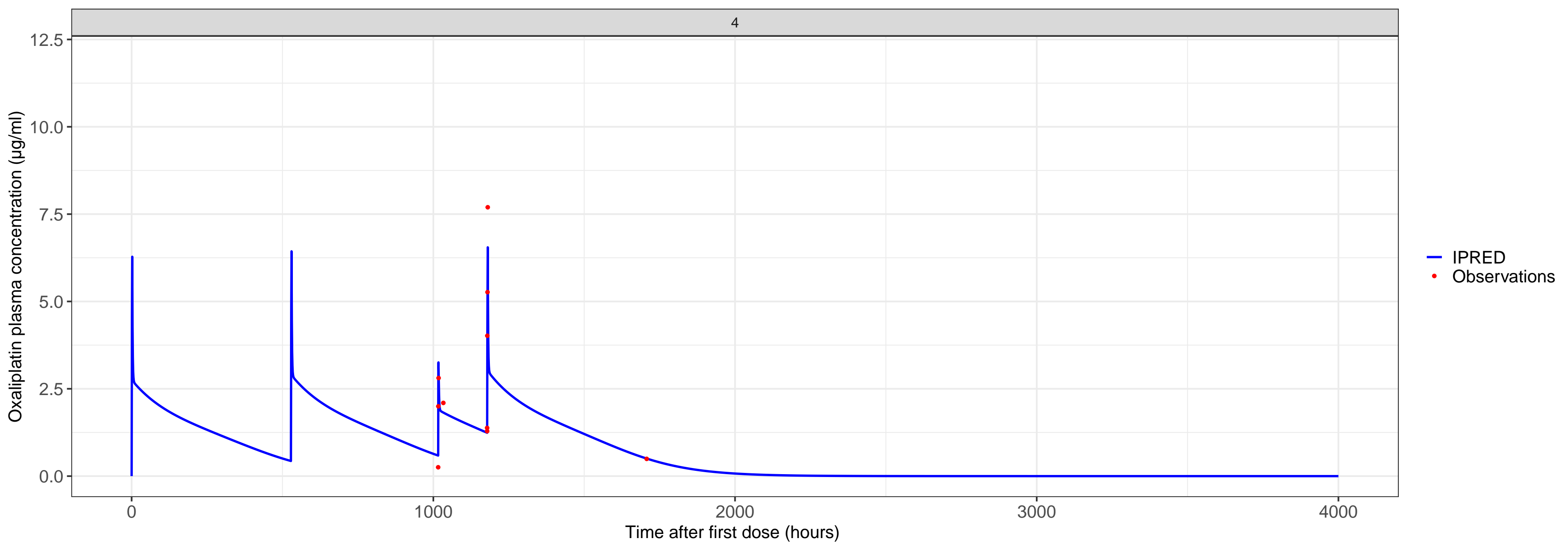

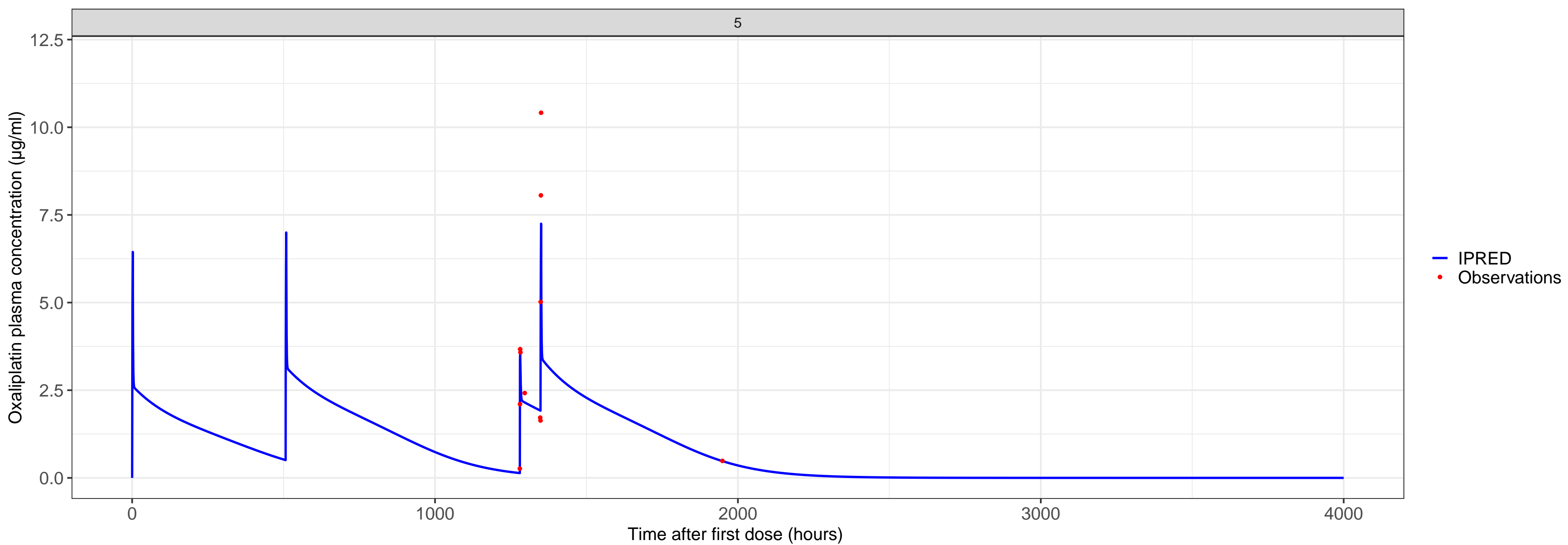

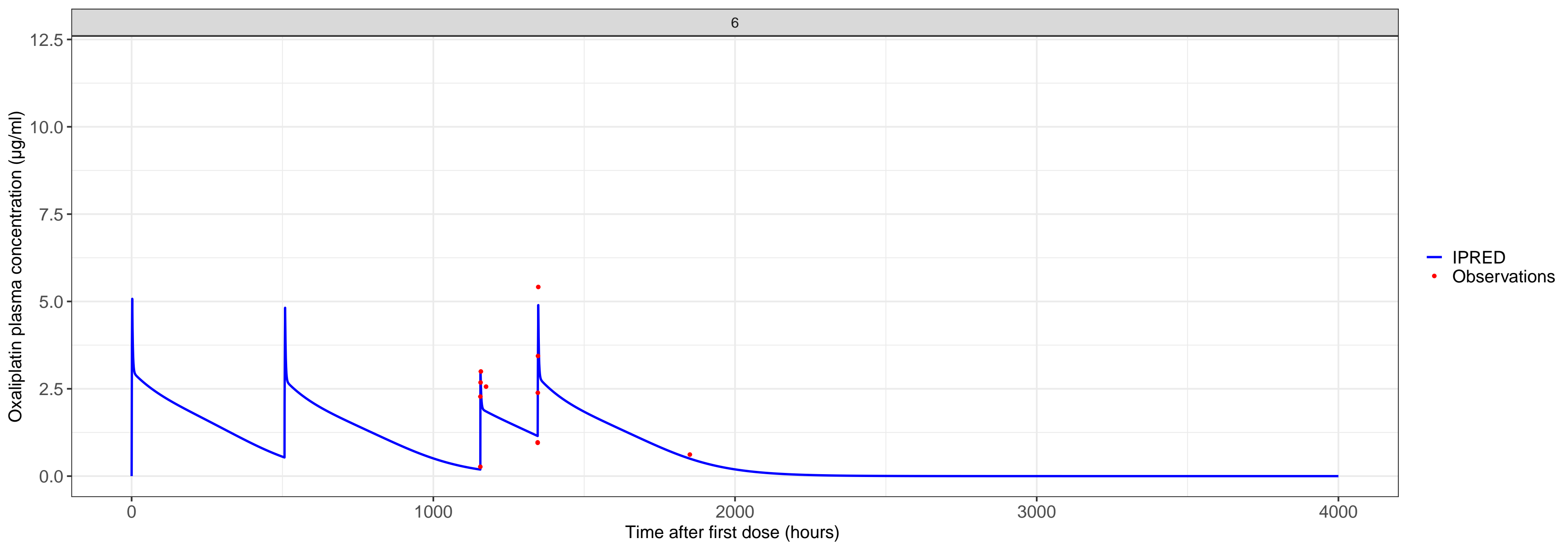

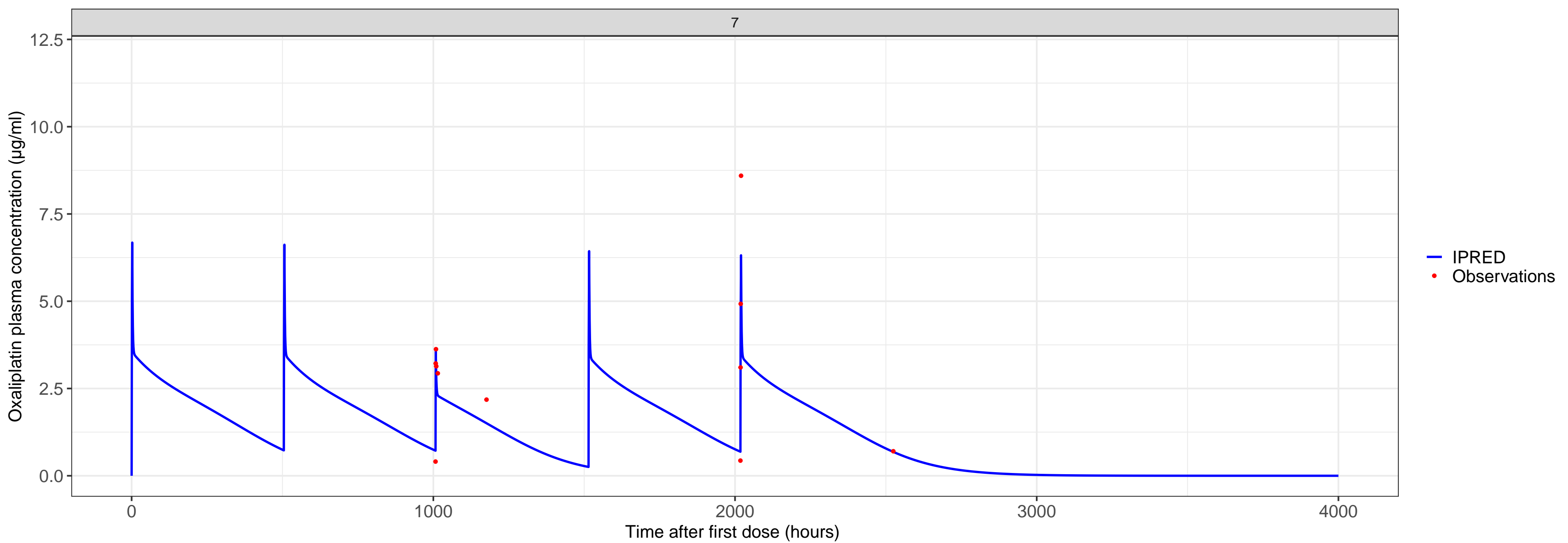

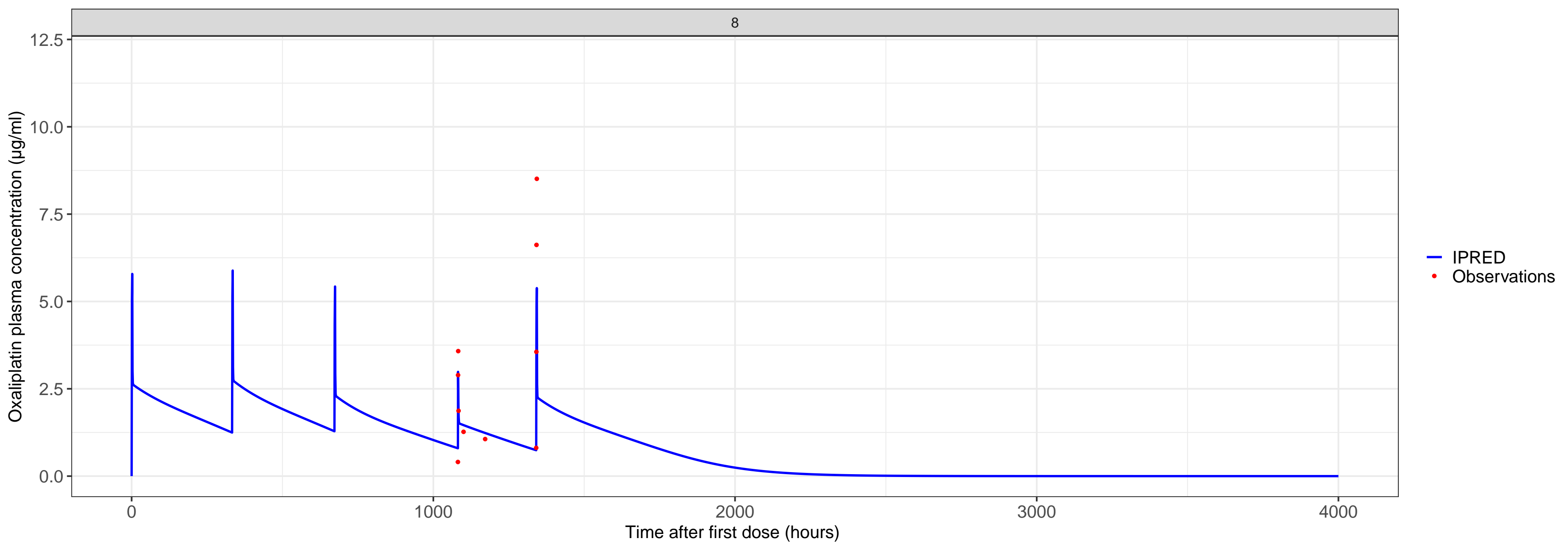

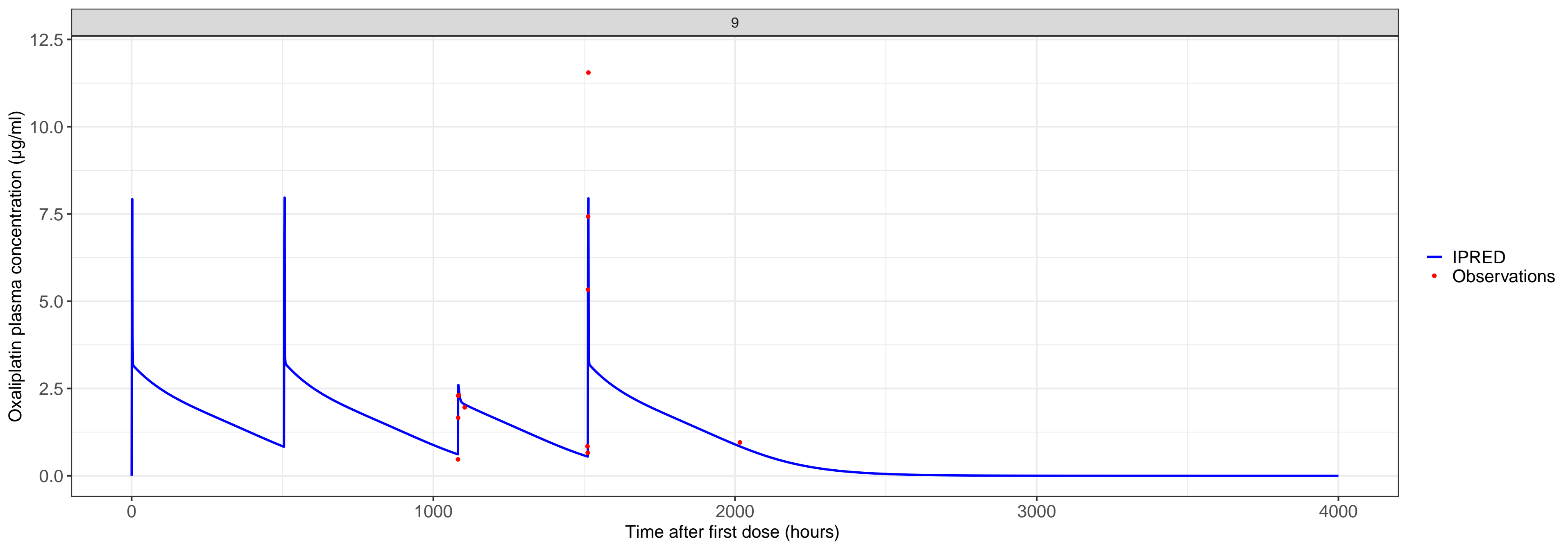

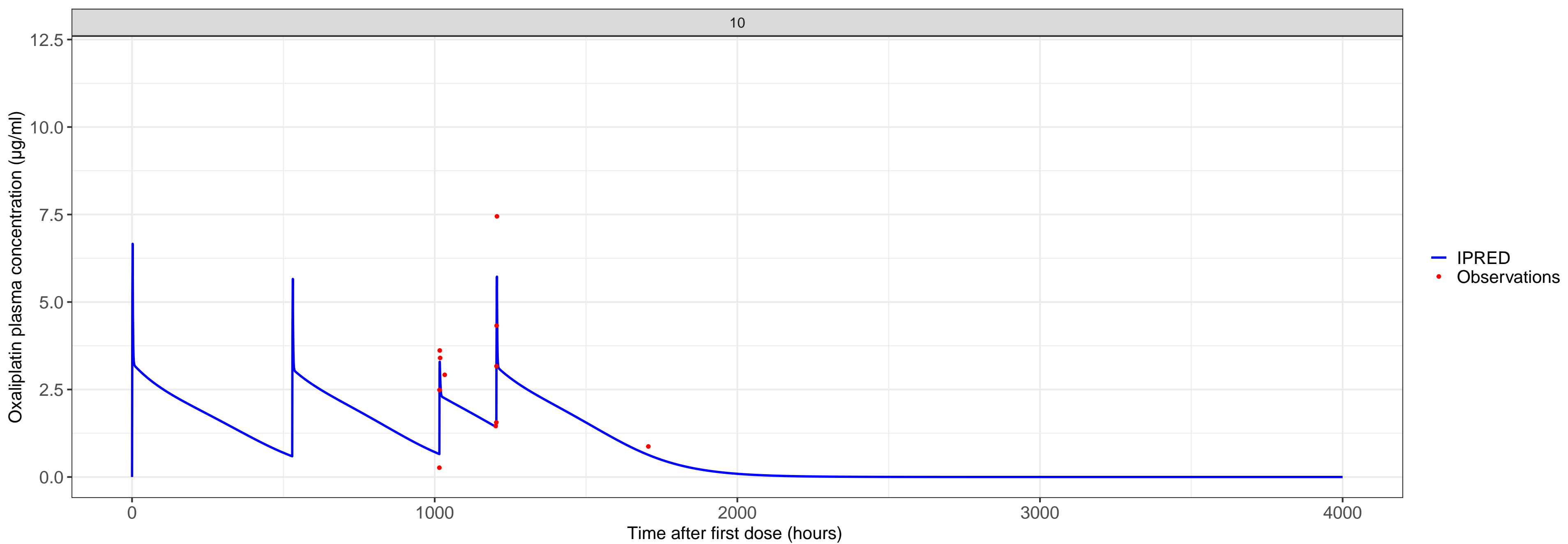

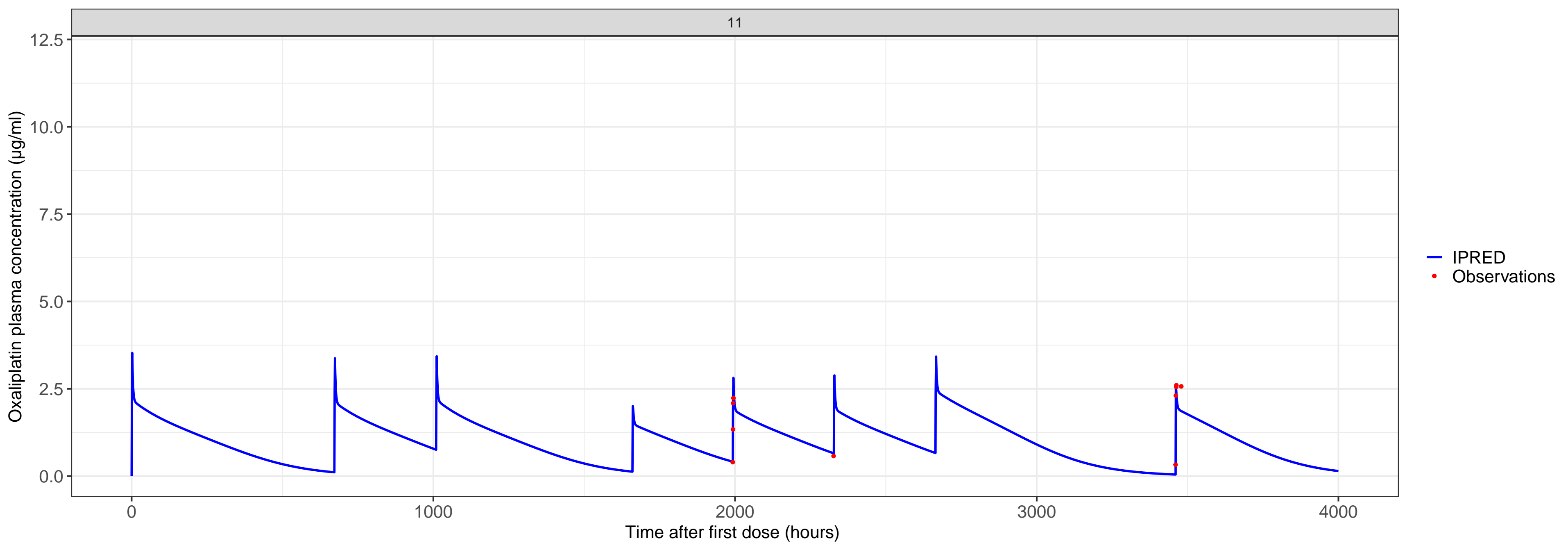

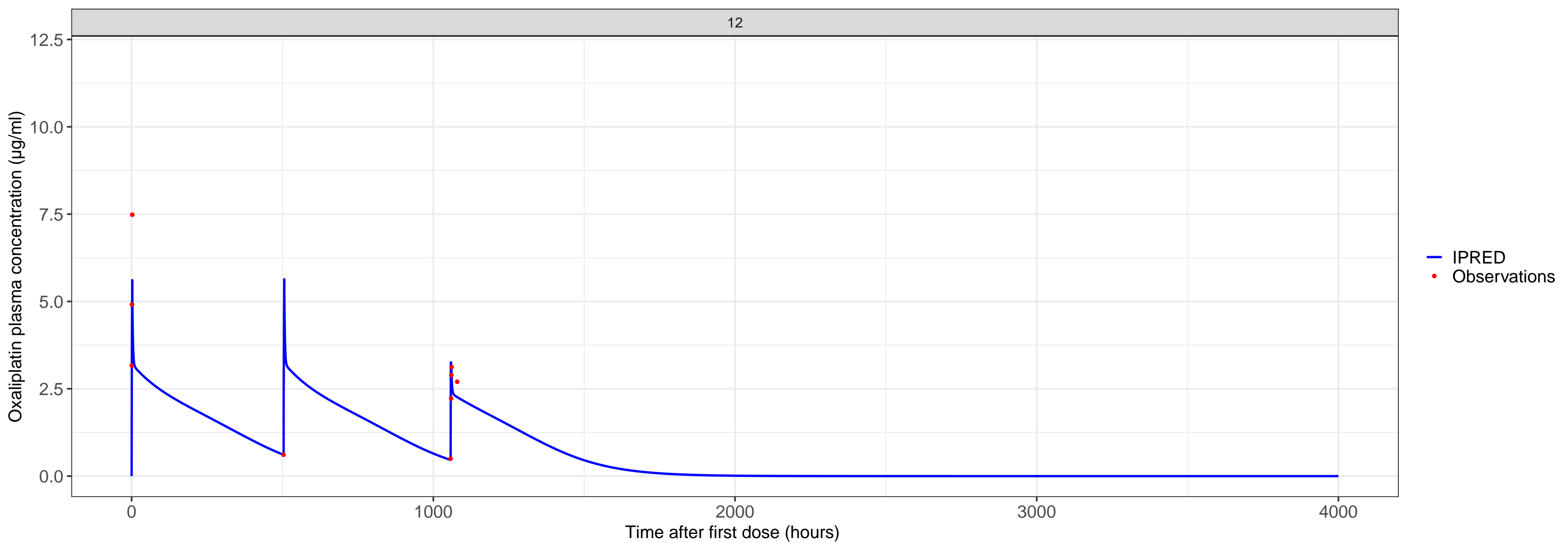

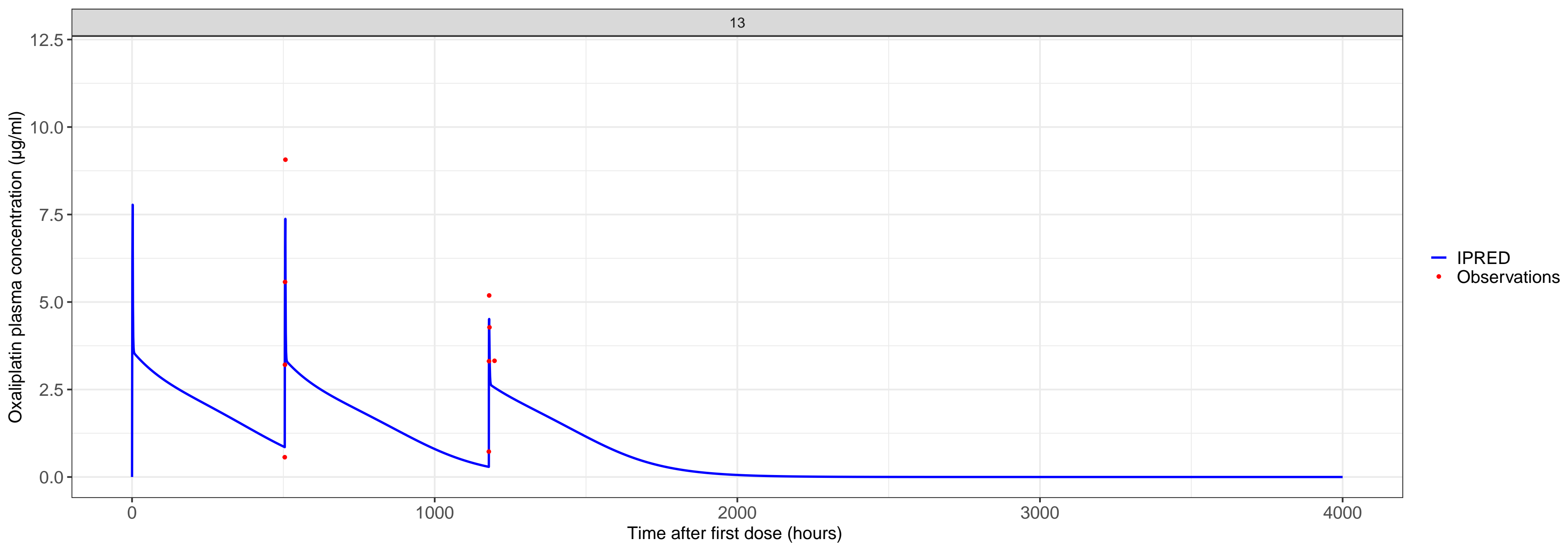

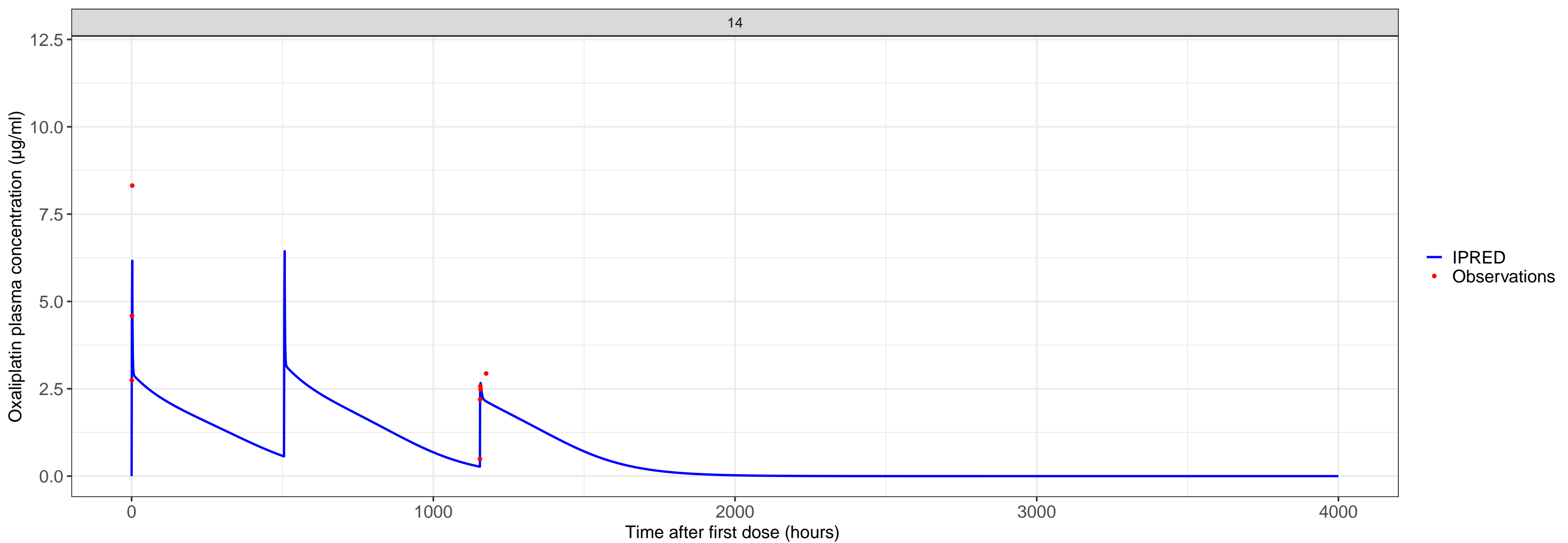

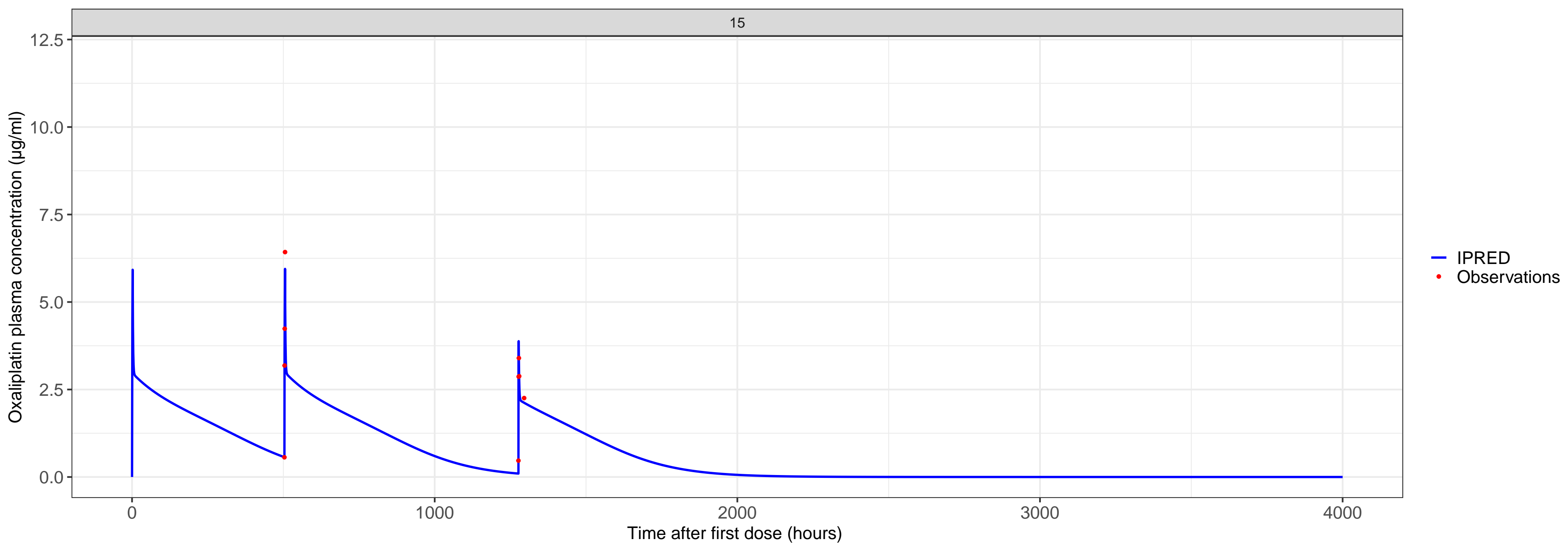

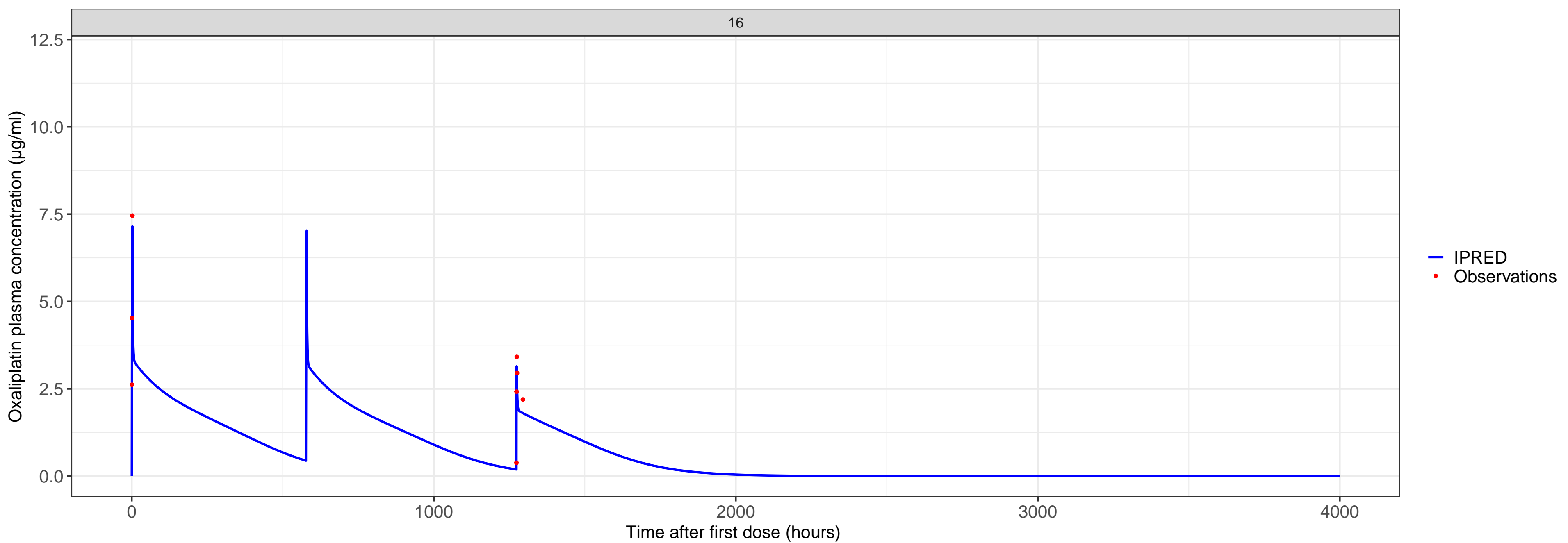

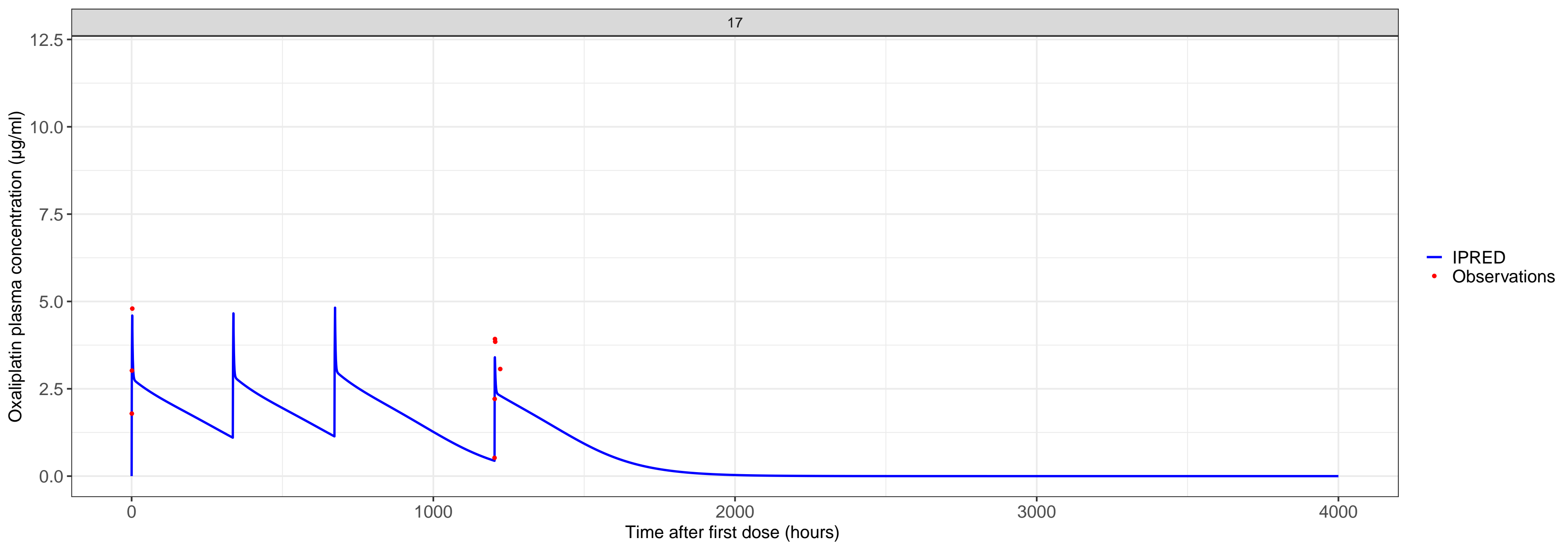

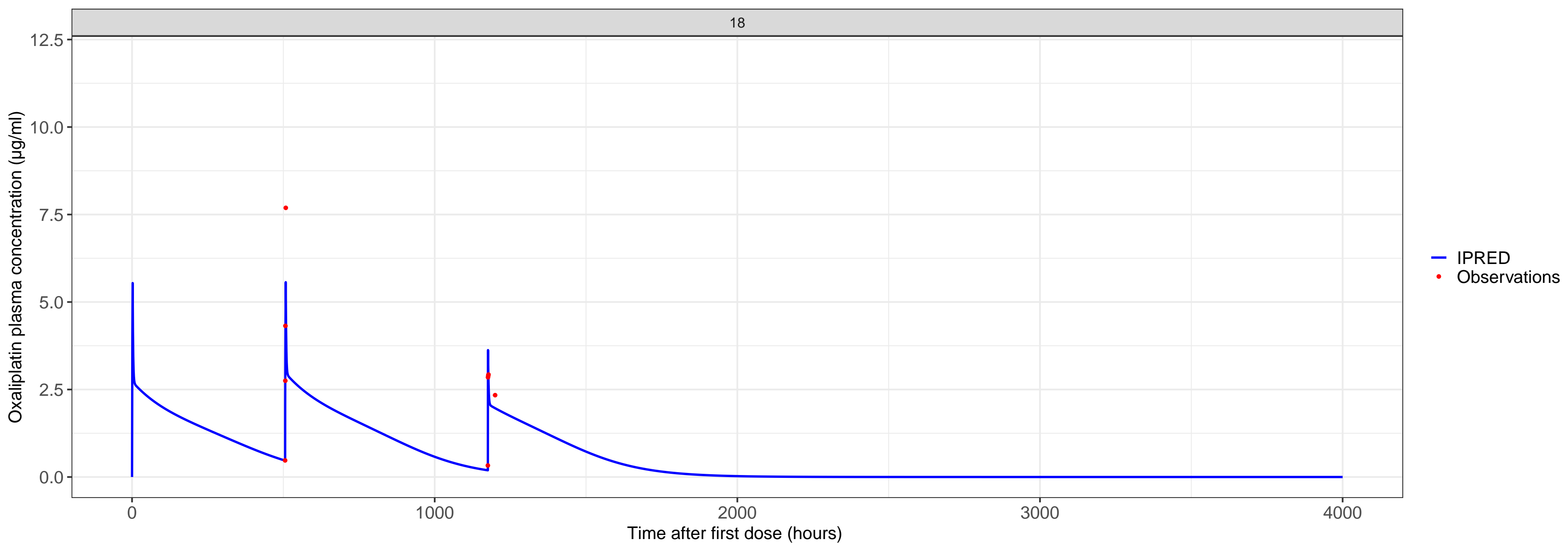

Supplement: Supplementary file 1 — (PDF 374 kb) [file 10434_2025_18874_MOESM1_ESM.pdf]

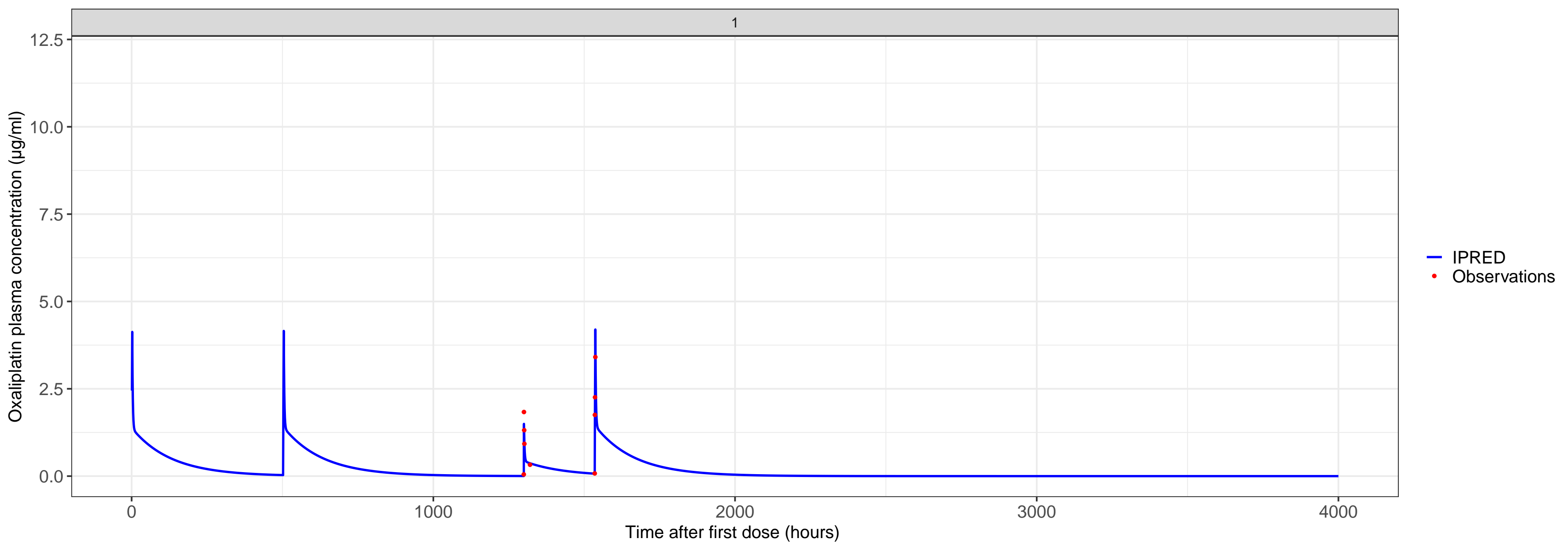

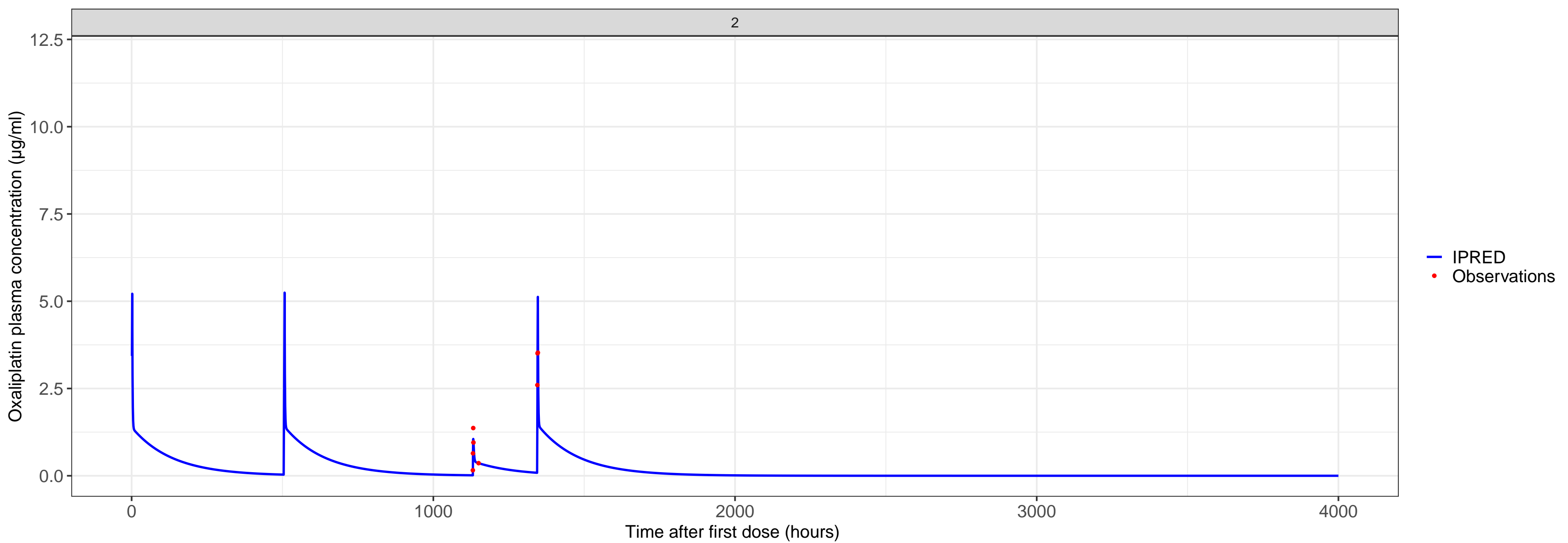

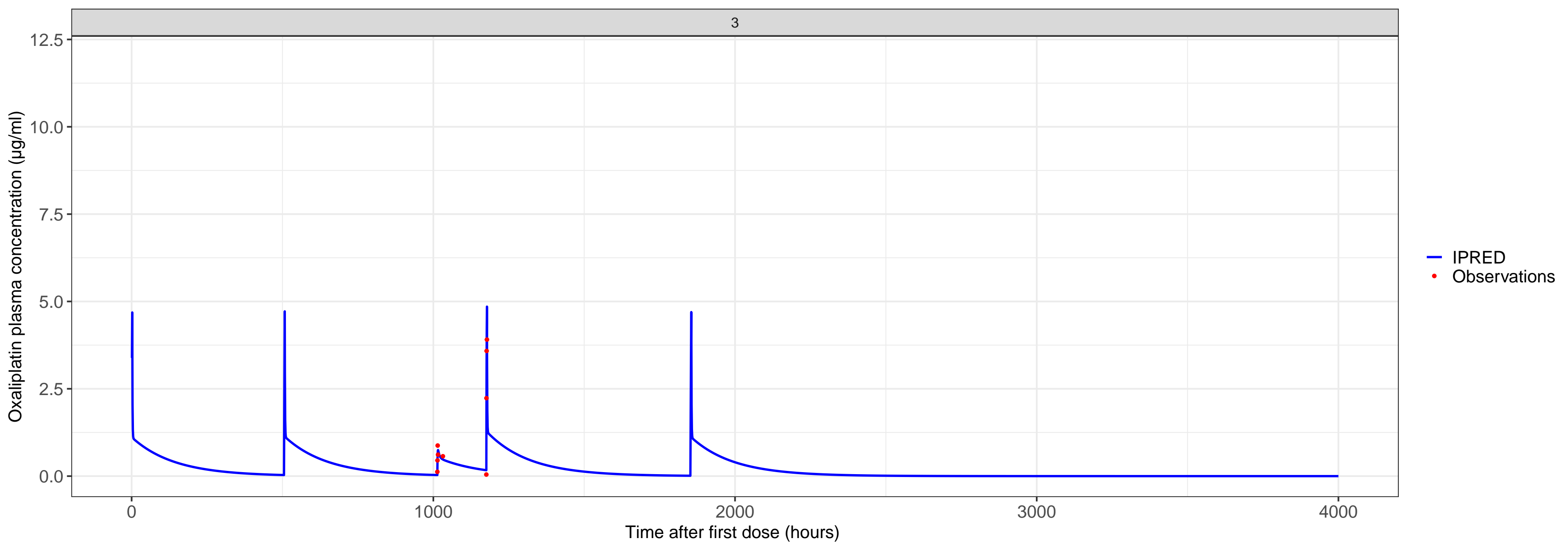

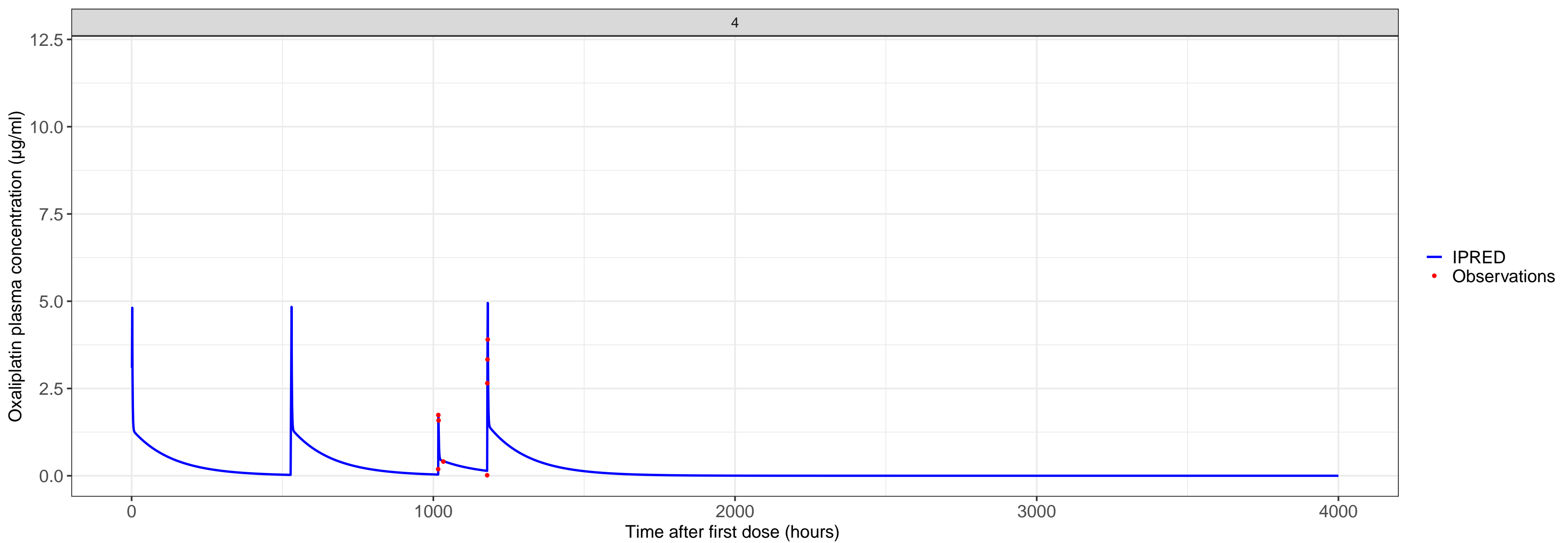

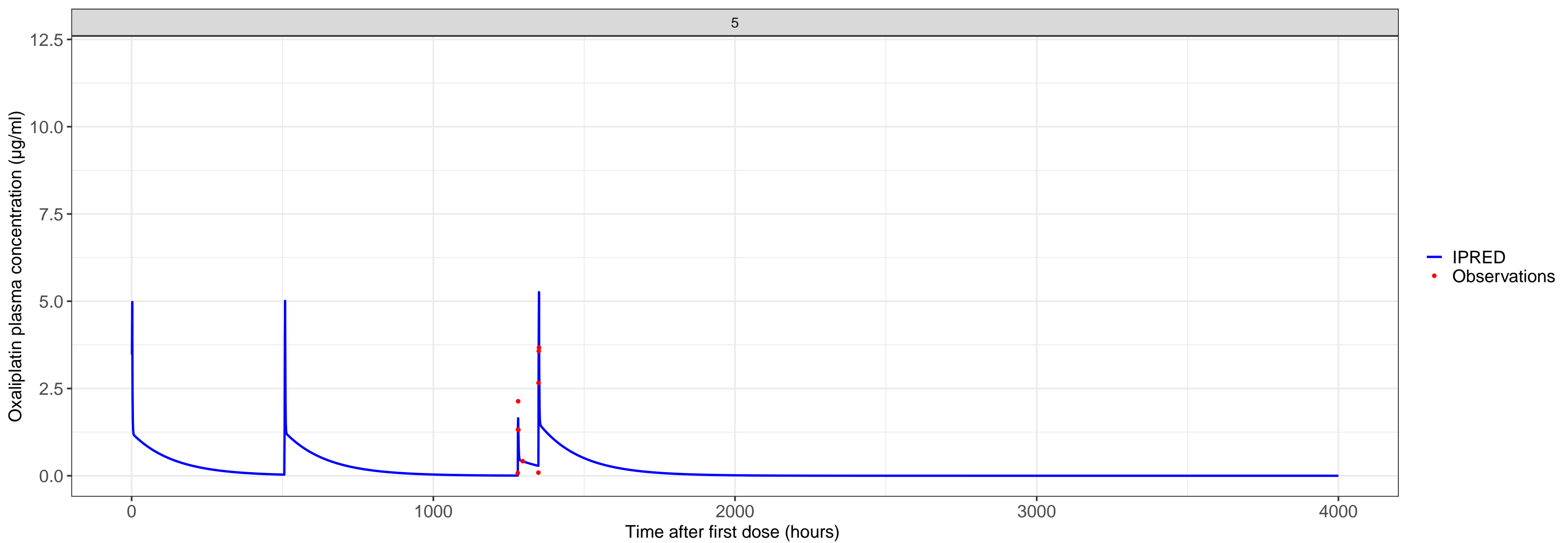

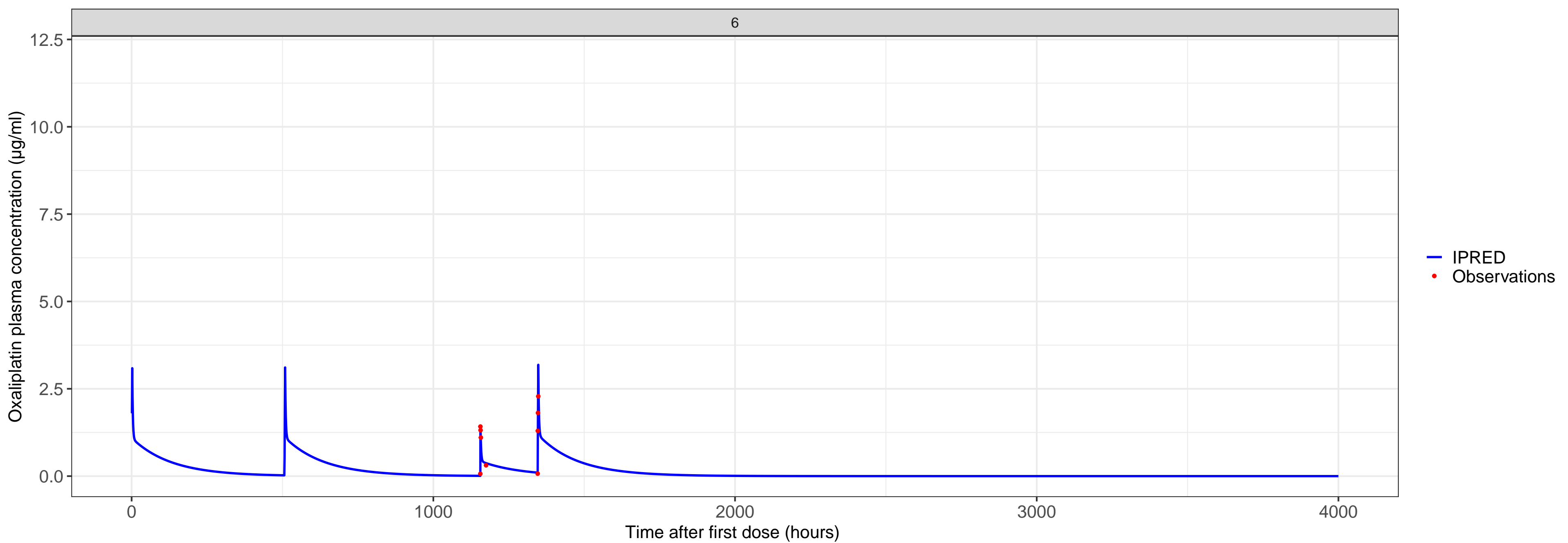

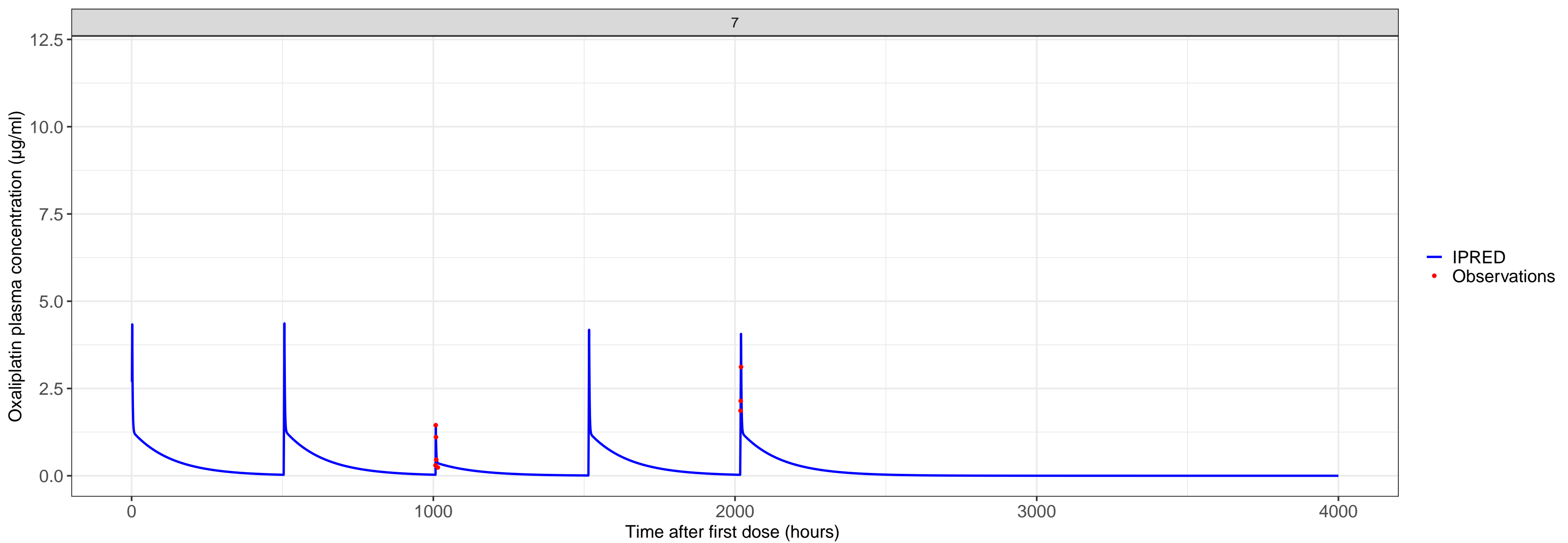

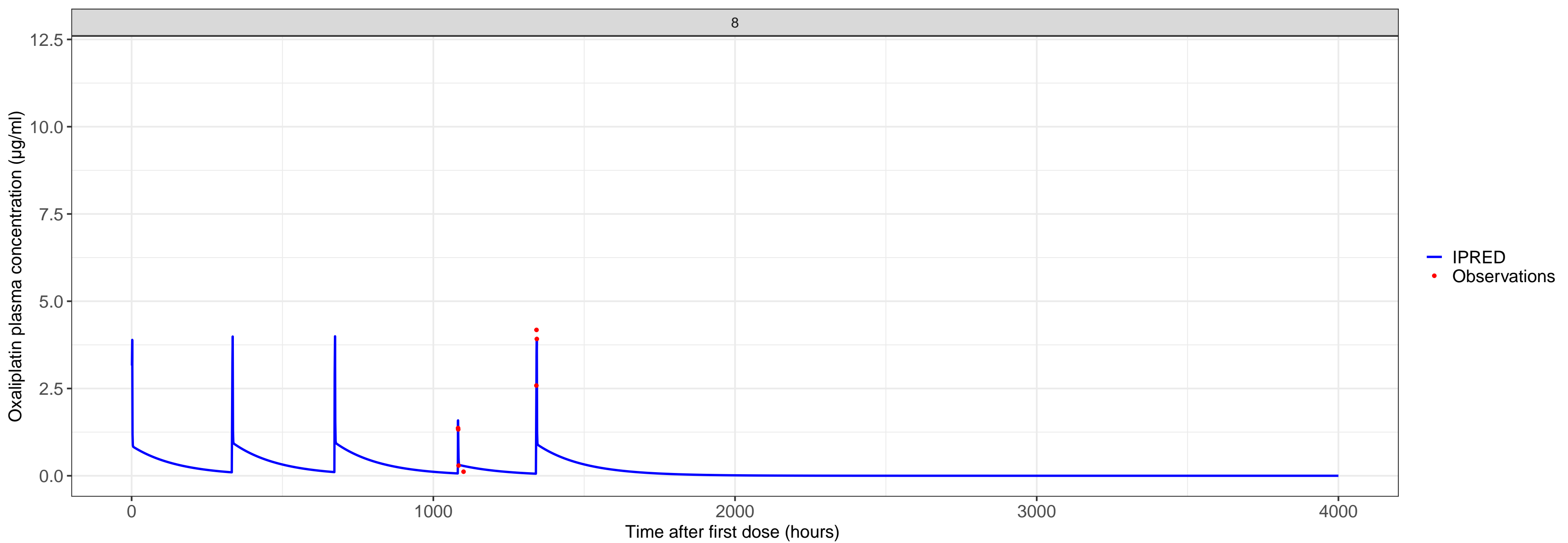

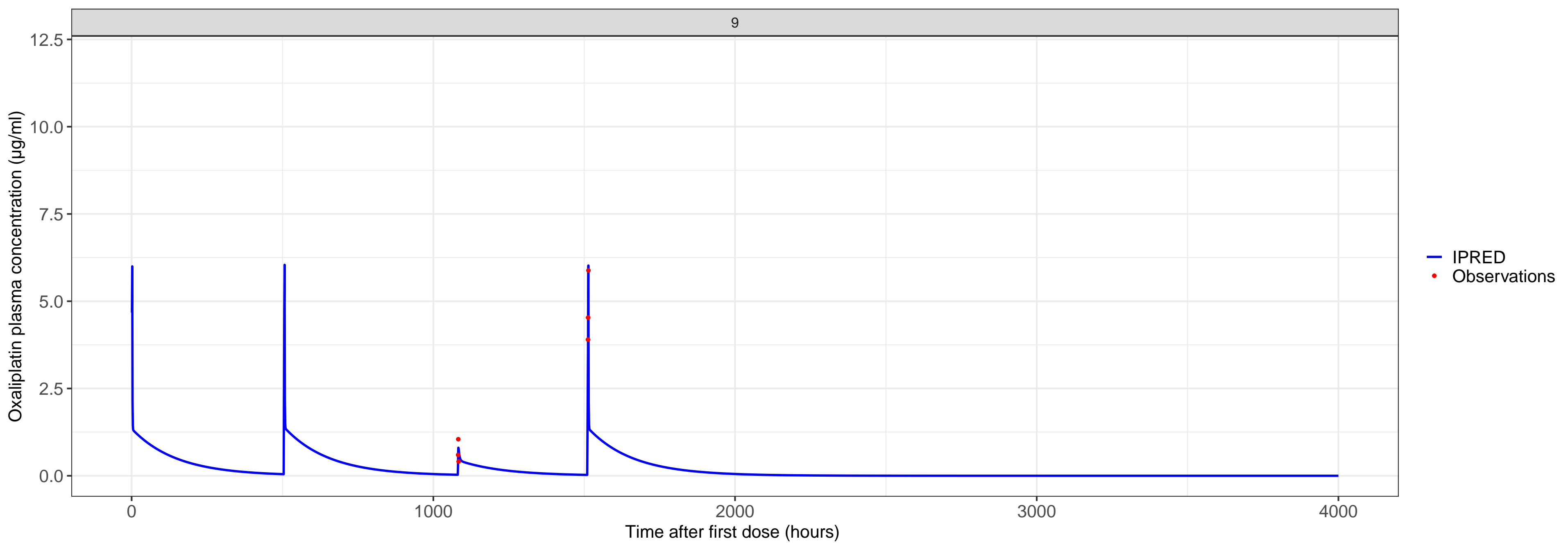

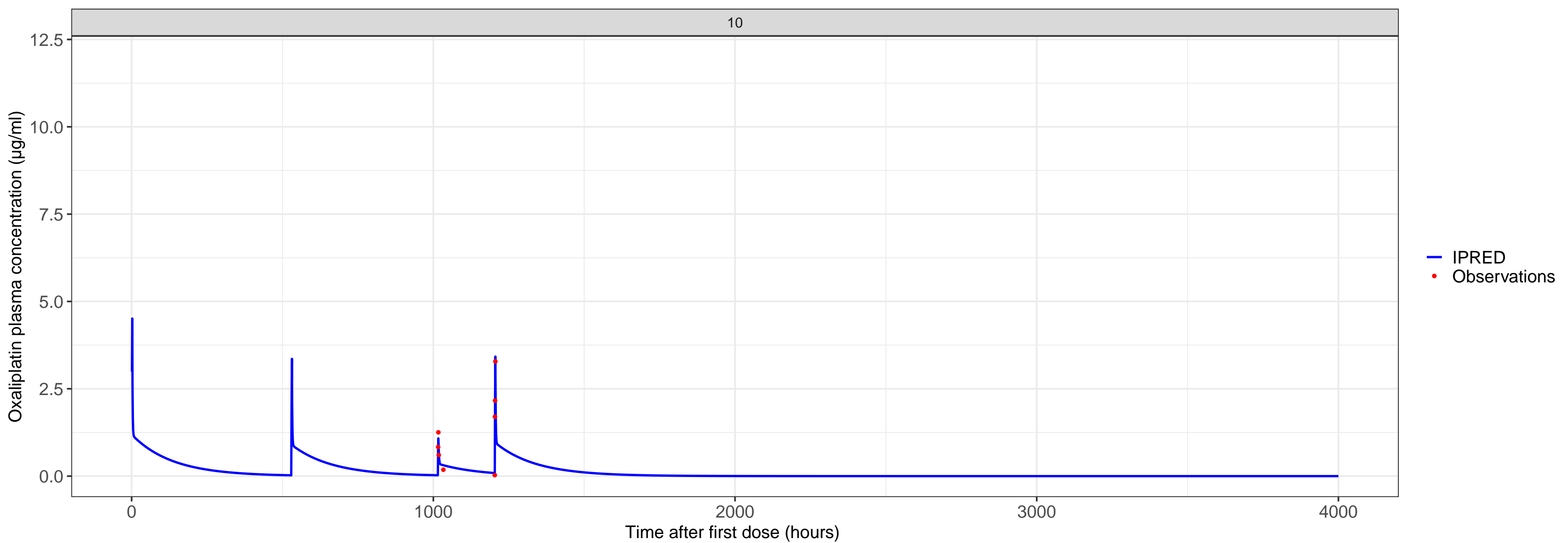

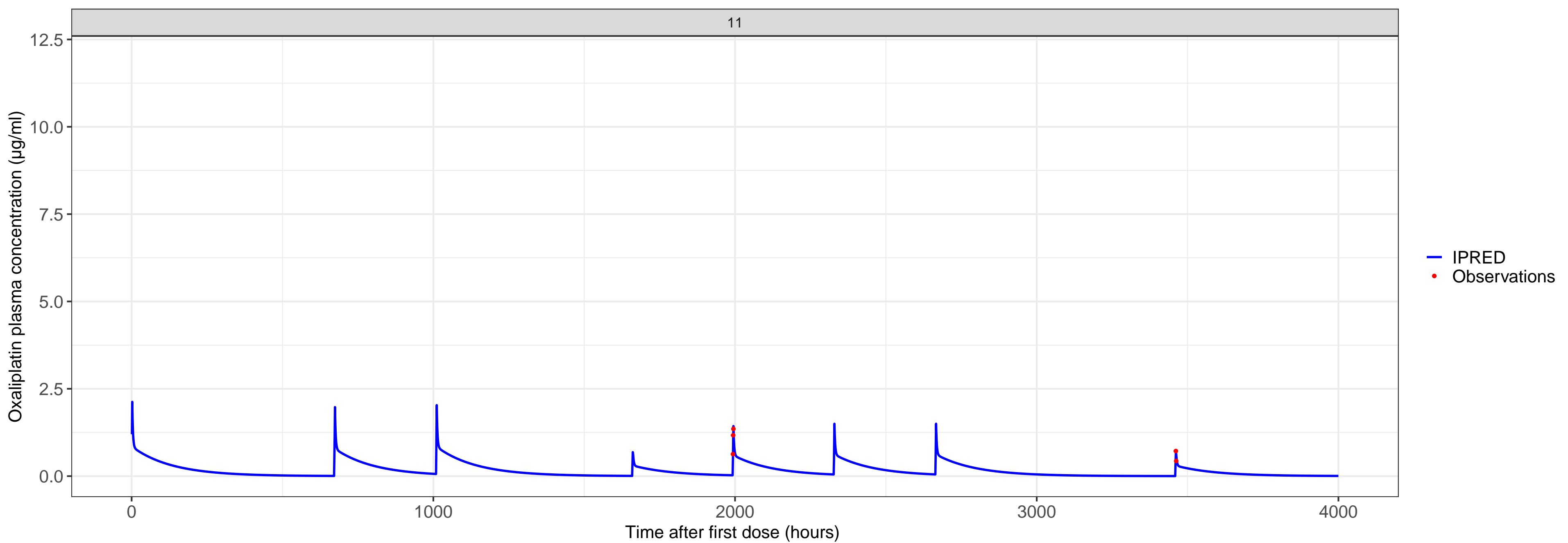

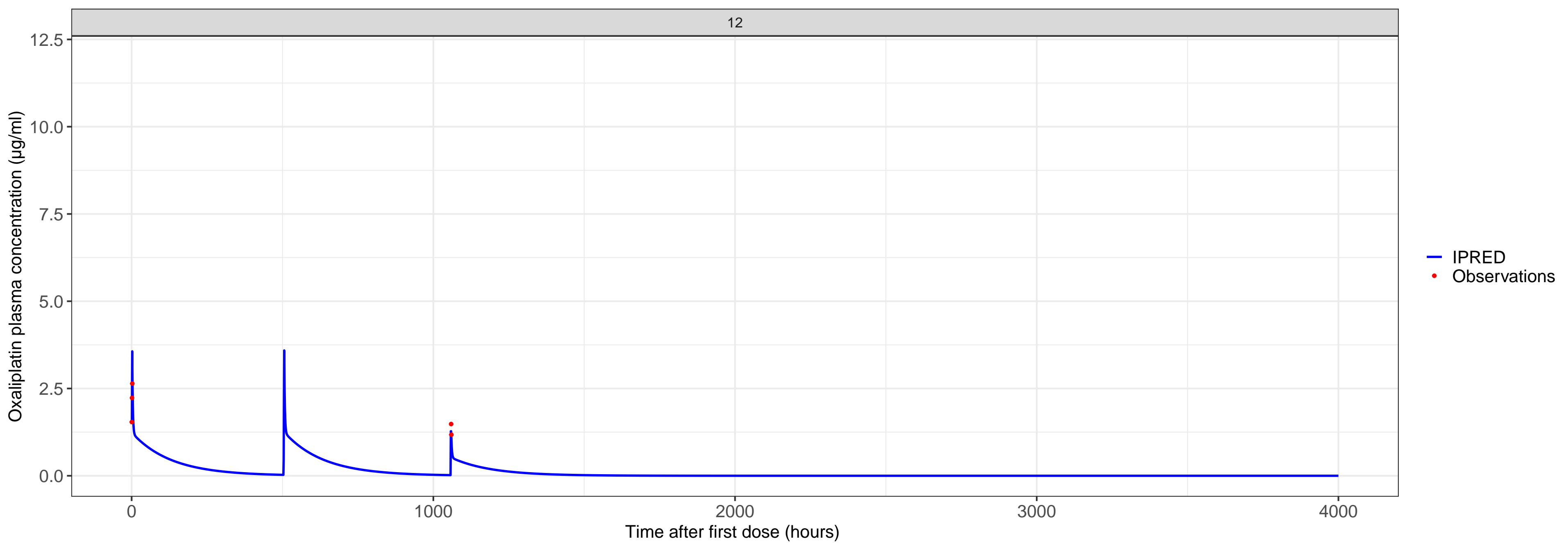

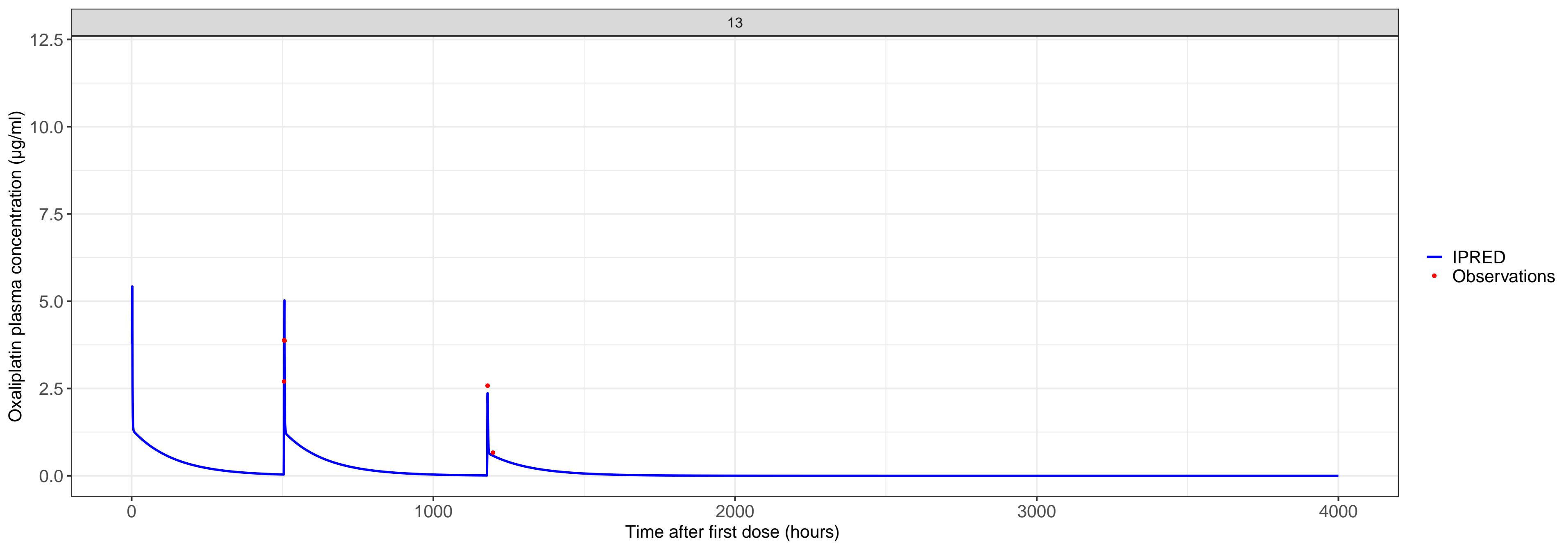

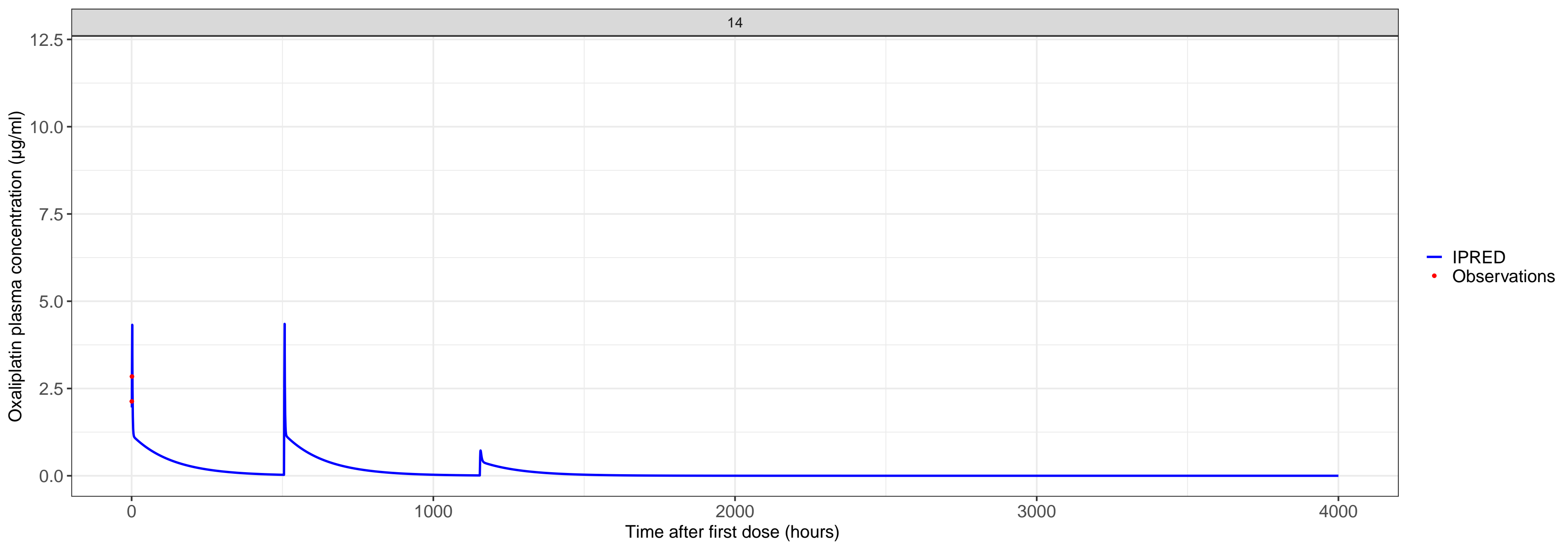

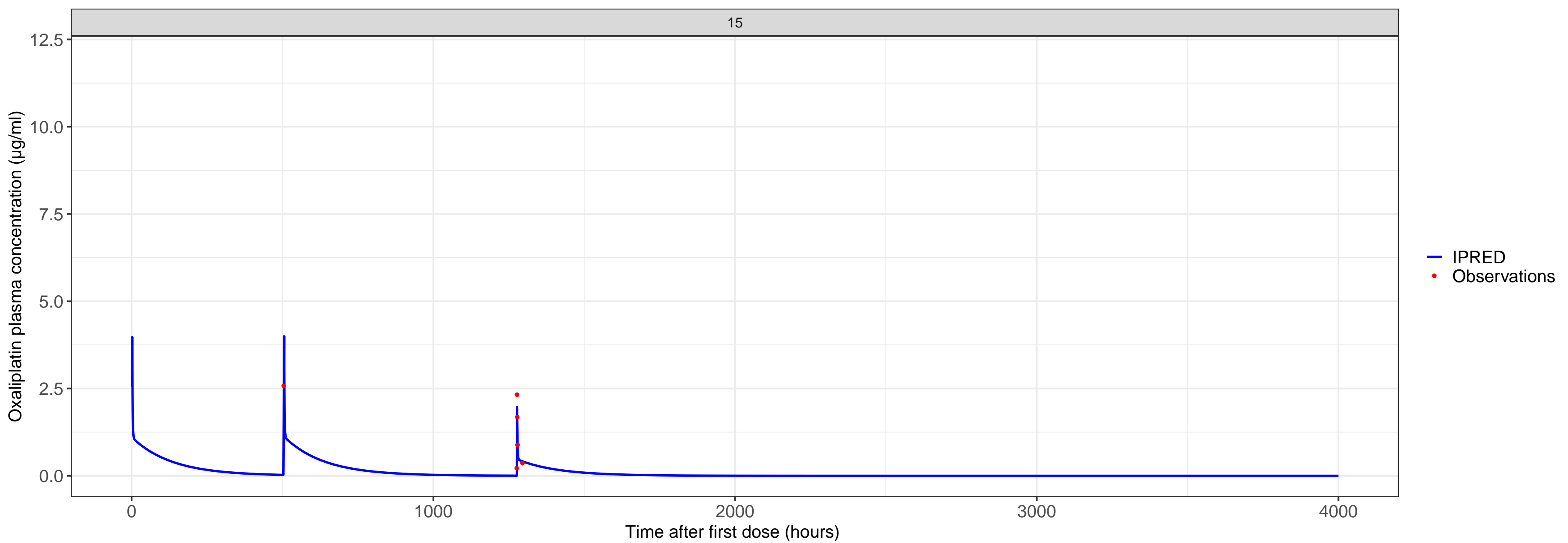

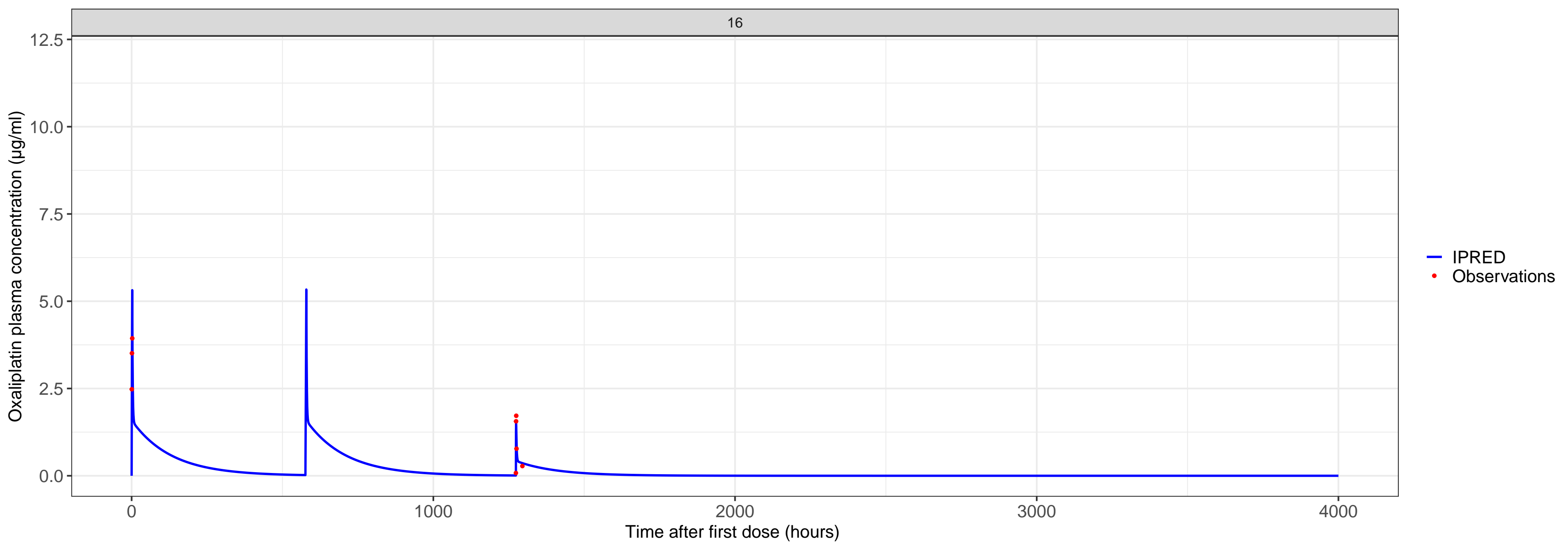

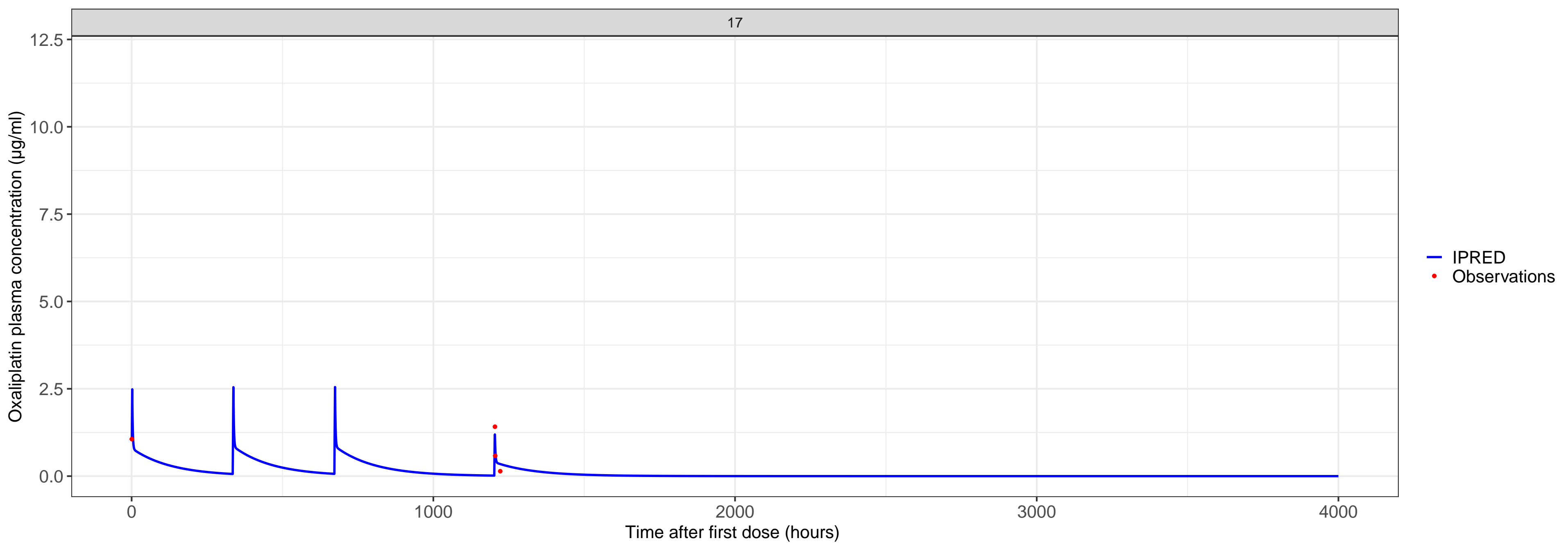

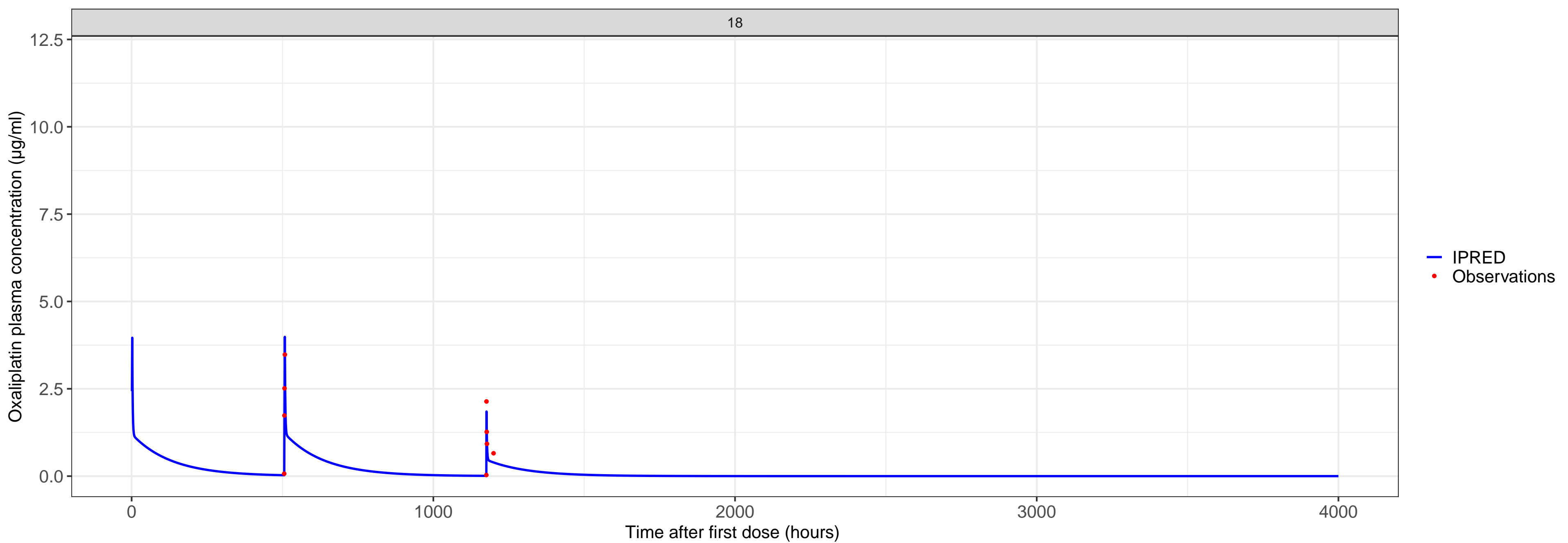

Supplement: Supplementary file 2 — (PDF 335 kb) [file 10434_2025_18874_MOESM2_ESM.pdf]
